# Benefits versus drawbacks of delaying surgery due to additional consultations in older patients with breast cancer

**DOI:** 10.1002/cnr2.1805

**Published:** 2023-03-21

**Authors:** Brian L. Egleston, Richard J. Bleicher, Carolyn Y. Fang, Thomas J. Galloway, Slobodan Vucetic

**Affiliations:** ^1^ Biostatistics and Bioinformatics Facility, Fox Chase Cancer Center Temple University Health System 333 Cottman Avenue Philadelphia Pennsylvania 19111 USA; ^2^ Department of Surgical Oncology, Fox Chase Cancer Center Temple University Health System 333 Cottman Avenue Philadelphia Pennsylvania 19111 USA; ^3^ Cancer Prevention and Control Program, Fox Chase Cancer Center Temple University Health System 333 Cottman Avenue Philadelphia Pennsylvania 19111 USA; ^4^ Department of Radiation Oncology, Fox Chase Cancer Center Temple University Health System 333 Cottman Avenue Philadelphia Pennsylvania 19111 USA; ^5^ Department of Computer and Information Sciences Temple University 1925 N. 12th St., 314 SERC Philadelphia Pennsylvania 19122 USA

**Keywords:** breast cancer, SEER‐Medicare, surgical delays, survival

## Abstract

**Background:**

Additional evaluations, including second opinions, before breast cancer surgery may improve care, but may cause detrimental treatment delays that could allow disease progression.

**Aims:**

We investigate the timing of surgical delays that are associated with survival benefits conferred by preoperative encounters versus the timing that are associated with potential harm.

**Methods and results:**

We investigated survival outcomes of SEER Medicare patients with stage 1–3 breast cancer using propensity score‐based weighting. We examined interactions between the number of preoperative evaluation components and time from biopsy to definitive surgery. Components include new patient visits, unique surgeons, medical oncologists, or radiation oncologists consulted, established patient encounters, biopsies, and imaging studies. We identified 116 050 cases of whom 99% were female and had an average age of 75.0 (*SD* = 6.2). We found that new patient visits have a protective association with respect to breast cancer mortality if they occur quickly after diagnosis with breast cancer mortality subdistribution Hazard Ratios [sHRs] = 0.87 (95% Confidence Interval [CI] 0.76–1.00) for 2, 0.71 (CI 0.55–0.92) for 3, and 0.63 (CI 0.37–1.07) for 4+ visits at minimal delay. New patient visits predict worsened mortality compared with no visits if the surgical delay is greater than 33 days (CI 14–53) for 2, 33 days (CI 17–49) for 3, and 44 days (CI 12–75) for 4+. Medical oncologist visits predict worse outcomes if the surgical delay is greater than 29 days (CI 20–39) for 1 and 38 days (CI 12–65) for 2+ visits. Similarly, surgeon encounters switch from a positive to a negative association if the surgical delay exceeds 29 days (CI 17–41) for 1 visit, but the positive estimate persists over time for 3+ surgeon visits.

**Conclusion:**

Preoperative visits that cause substantial delays may be associated with increased mortality in older patients with breast cancer.

## 1 INTRODUCTION

Timeliness of breast cancer treatment is a concern.^1^, ^2^, ^3^ The timing of definitive treatment is subject to many factors, including preoperative evaluation components which include consultations, imaging, and biopsies,^4^ care transfers between institutions,^5^ and multidisciplinary preoperative evaluations.^6^, ^7^ Preoperative consultations with a plastic surgeon to discuss immediate breast reconstruction may also cause delays.

Longer times between diagnosis and surgery for both invasive breast cancer^8^ and ductal carcinoma in situ (DCIS)^9^ are associated with detrimental survival and increased risk of disease progression. Relative overall mortality for patients having invasive breast cancer increases by 9% per month of delay,^8^ while the risk of invasive disease progression for patients with DCIS increases by 13% per month of delay.^9^ The delay‐related relative increase in invasion and mortality is the end result of a potentially long growth interval that occurs before a tumor is diagnosed.^10^

Clinical evaluation components (e.g., surgical or medical oncology consultations) in patients with breast cancer provide potential benefit by optimizing treatment. In addition, patients often seek second opinions,^11^ and those opinions could be helpful. However, scheduling and attending extra evaluations and clinical consultations could delay time until surgery. This delay may allow disease progression and a consequent reduction in survival.

In this paper, we attempt to determine when a delay is worthwhile since the delay may allow for additional provider visits that confer a benefit. Related, we try to estimate the number of days after biopsy that extra consultations may no longer be helpful.

## 2 METHODS

We used Surveillance Epidemiology and End Results (SEER) database linked to Medicare claims (https://seer.cancer.gov/registries/). As a population based registry, SEER‐Medicare data is generalizable to the majority of individuals in the United States over 65 years old. The SEER‐Medicare database not only has detailed clinical and survival information, but also contains comprehensive claims data with dates regarding physician visits and procedures. This allowed us to investigate the relationship of survival outcomes with the number and type of visits in the interval between biopsy and surgery. We hypothesized that there would be interactions between the types of consultation in the preoperative interval and delays on outcomes. A limitation of SEER‐Medicare is that the most recent cancer cases are not included since there is a time lag of several years before data is released. However, few other datasets have detailed information needed to explore this topic. We included surgically treated breast cancer cases that were diagnosed from 1992 to 2013 with follow‐up through 2014. We included all individuals at least 66 years old with Medicare Parts A and B coverage for whom breast cancer was their first lifetime cancer. Those in Medicare Part C (i.e., managed care) were excluded. We excluded those who had neoadjuvant chemotherapy or radiotherapy.

We defined delay as months from first biopsy (in the month or prior month of diagnosis) to date of definitive surgical procedure after excluding those who had neoadjuvant chemotherapy, as described previously.^8^, ^12^ We defined a month as 30.44 days (365.25/12), and we examined intervals between biopsy and surgery of 1 to 180 days. Our data indicated that effects changed at approximately one calendar month post biopsy. We examined the number of procedures and office visits on different days within the interval between the diagnostic biopsy and definitive procedure. We did not count multiple visits of the same type on the same day and did not include the end points (i.e., biopsy or surgical date) of the interval. We categorized procedures and grouped extreme numbers.

We used Medicare claims International Classification of Disease (ICD‐9) and Healthcare Common Procedure Coding System (HCPCS) codes to determine type of visit (e.g., established or new patient visit) or procedure. We determined physician specialty using health care financing administration specialty codes. We evaluated the number of unique physicians by applying the NCI crosswalk to link physician identifiers over time. All codes are specified in the supporting information.

We investigated SEER‐captured breast cancer‐specific mortality using Fine and Gray regressions,^13^ and overall survival using Cox regressions. We controlled for baseline differences among encounter groups using propensity score based weighting.^14^ We estimated propensity scores separately for each component. We included the following variables in the propensity score model: age at diagnosis, Charlson comorbidity index,^15^ Elixhauser score,^16^ race, sex, SEER region of the country, marital status, metropolitan area size, tumor size, diagnosis year, whether nodes were examined, number of positive nodes, histology, tumor grade, ER/PR positivity, HER‐2 positivity, tumor sequence, and AJCC stage. Therapeutic variables included mastectomy versus lumpectomy, reconstruction, adjuvant chemotherapy, and adjuvant radiation therapy. Patient ZIP code‐level variables included the percent of the population with less than a high school education or living below the poverty line. We used restricted cubic splines^17^ for continuous variables and robust standard errors.^14^

In propensity‐score base weighted regressions, we included main effects for procedure group (categorical variable) and treatment delay (continuous variable) and their interactions. We set the reference (subdistribution hazard ratio [sHR] = 1 or log sHR = 0) to be the survival effect under 0 relevant encounters and no treatment delay. We present cumulative incidence and survival curves by combining baseline functions with regression parameter estimates. For ease of presentation, we plotted four curves assuming zero or the maximum number of encounters, and delay of 0.5 or 3 months. We used STATA and a nominal *p* < .05 defined statistical significance.

## 3 RESULTS

**FIGURE 1:**
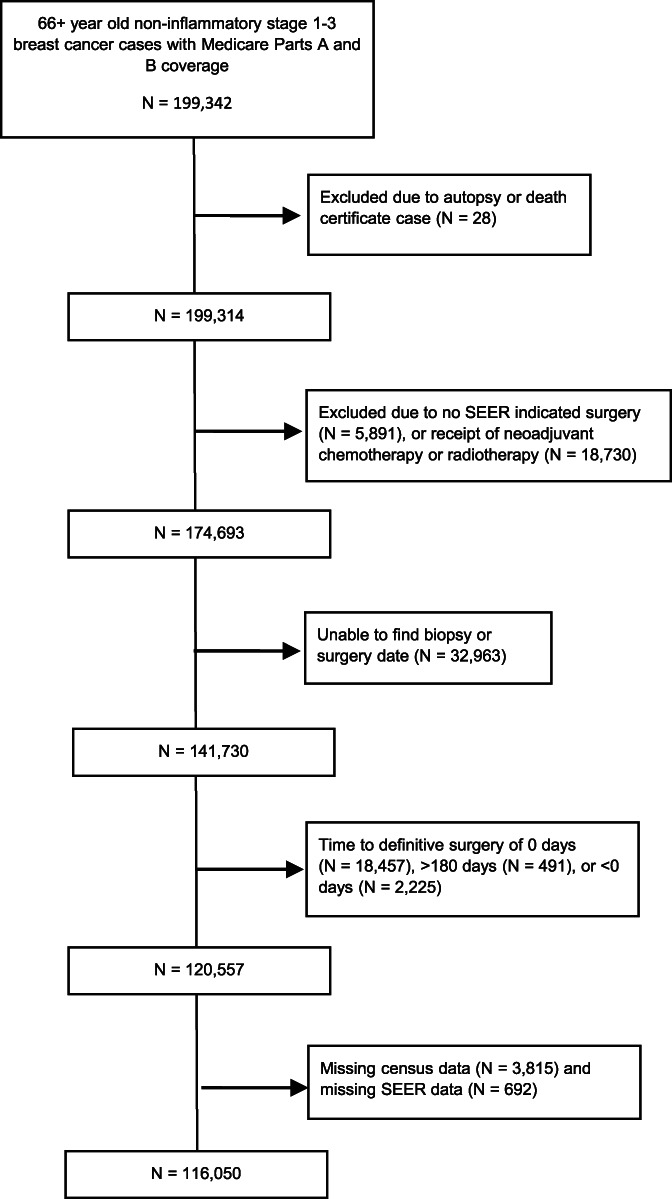
STROBE diagram enumerating cohort inclusions and exclusions.

Figure 1 describes inclusions and exclusions. The most common reasons for exclusions were that patients received neoadjuvant chemotherapy (that necessarily delays surgery) or that a biopsy date was not found. Table 1 shows cohort demographic and clinical characteristics. The mean delay was 26.3 days (Standard Deviation = [*SD*] 19.6), and the average age was 75 (*SD* = 6). 88.6% of the sample was of European Ancestry, 6.5% were of African Ancestry, and 2.1% were Asian. Notably, a quarter of the sample had delays longer than 34 days. Since SEER is a population based registry, Table 1 statistics represent the characteristics of the population of Medicare Parts A and B beneficiaries in the SEER areas.

**TABLE 1:** Demographic and clinical characteristics of the sample.

Characteristic	Mean/Median or *N* (%)
*Days delay* (*time from first biopsy to surgery*)	
Mean (*SD*)	26.32 (19.60)
Median (Q1, Q3)	21.0 (14.0, 34.0)
*Age at diagnosis*	
Mean (*SD*)	75.02 (6.18)
Median (Q1, Q3)	74.0 (70.0, 79.0)
*Background*	
European ancestry	102 851 (88.6%)
African ancestry	7519 (6.5%)
Other or unknown	2101 (1.8%)
Asian	2408 (2.1%)
Hispanic	1171 (1.0%)
*Marital status*	
Not married	61 804 (53.3%)
Married	54 246 (46.7%)
*Charlson comorbidity Index*	
Mean (*SD*)	0.65 (1.07)
Median (Q1, Q3)	0.0 (0.0, 1.0)
*Elixhauser score*	
Mean (*SD*)	1.90 (5.30)
Median (Q1, Q3)	0.0 (0.0, 3.0)
*% of zip code with less than a high school education*	
Mean (*SD*)	15.19 (10.39)
Median (Q1, Q3)	12.7 (7.5, 20.5)
*% of zip code living below poverty line*	
Mean (*SD*)	12.28 (8.51)
Median (Q1, Q3)	9.9 (6.0, 16.7)
*Census region of the United States*	
Northeast	21 059 (18.1%)
Midwest	18 611 (16.0%)
West	50 798 (43.8%)
South	25 582 (22.0%)
*Type of metropolitan area*	
Big Metro	63 236 (54.5%)
Metro	34 109 (29.4%)
Urban	6685 (5.8%)
Less urban/rural	12 020 (10.4%)
*SEER defined sex*	
Male	1070 (0.9%)
Female	114 980 (99.1%)
*Sequence number of cancer* *	
Mean (*SD*)	1.08 (0.30)
Median (Q1, Q3)	1.0 (1.0, 1.0)
*Tumor size* (*mm*)	
Mean (*SD*)	18.79 (19.06)
Median (Q1, Q3)	15.0 (10.0, 23.0)
*Year of breast cancer diagnosis*	
1992–1999	17 817 (15.4%)
2000–2009	68 719 (59.2%)
2010–2013	29 514 (25.4%)
*Number of lymph nodes positive*	
Mean (*SD*)	0.87 (2.70)
Median (Q1, Q3)	0.0 (0.0, 0.0)
*Histology*	
Ductal	100 485 (86.6%)
Lobular	12 953 (11.2%)
Other or unknown	2612 (2.3%)
*Grade*	
Well	27 608 (23.8%)
Moderate	50 384 (43.4%)
Poor	28 353 (24.4%)
Undifferentiated	1104 (1.0%)
Unknown	8601 (7.4%)
*ER or PR positive*	
Negative	24 180 (20.8%)
Positive	91 870 (79.2%)
*HER2 positive*	
Negative	113 413 (97.7%)
Positive	2637 (2.3%)
*AJCC stage*	
Stage 1	68 528 (59.1%)
Stage 2	40 243 (34.7%)
Stage 3	7279 (6.3%)
*Type of surgery*	
Lumpectomy only	60 276 (51.9%)
Mastectomy	55 774 (48.1%)
*Nodes examined*	
No	2029 (1.7%)
Yes	114 021 (98.3%)
*Reconstructive surgery*	
No	112 267 (96.7%)
Yes	3783 (3.3%)
*Adjuvant chemotherapy*	
No adjuvant	88 506 (76.3%)
Adjuvant	27 544 (23.7%)
*Adjuvant radiotherapy*	
No adjuvant	53 310 (45.9%)
Adjuvant	62 740 (54.1%)

*Note*: Q1, Q3 = Interquartile range. (*n* = 116 050).

Abbreviation: SD, standard deviation.

* Sequence of cancer refers to the order of all cancers that the first breast cancer occurred. For example, if someone had a single prior unrelated cancer, their first breast cancer would be their second lifetime cancer. We included cancer survivors with prior non‐breast cancers to improve generalizability. Our definitions for demographic and presentation variables are based on the SEER program definitions.

Table 2 provides descriptive statistics of the number of unique (i.e., different) encounters with the health care system that patients had in the interval between biopsy and surgery. On average, patients had 3.85 (*SD* = 3.35) health care encounters between biopsy and surgery. However, relatively few of these encounters were with cancer specialists or related to biopsy or imaging studies. For example, the average number of unique medical oncologists (i.e., different medical oncologists) consulted was 0.16 (*SD* = 0.38), which implies that relatively few patients visited a medical oncologist in the time between biopsy and surgery. Some patients consulted 1 or more medical oncologists, while most visited 0 medical oncologists, resulting in a mean (i.e., average) number consulted of 0.16. More patients consulted a surgeon in the preoperative interval, with the mean being 0.75 (*SD* = 0.56). The average number of unique radiation oncologist visits (mean 0.11 *SD* = 0.32), biopsies (0.16 *SD* = 0.42), and imaging studies (0.48 *SD* = 0.83) per patient was relatively low. The average number of established provider visits (mean 1.36 *SD* = 1.53) was larger than of new patient visits (0.86 *SD* = 0.95); established visits are those for which a patient previously has had a consultation with a physician. The second column of Table 3 provides counts of the number of encounters or procedures, which adds additional information to Table 2. The counts in Table 3 demonstrate that the absolute number of cases with multiple encounters of all types was large, even if the proportion was low. In supplemental Tables 1–12, we show descriptive statistics by selected procedure groupings before and after adjustment by propensity score based weighting.

**TABLE 2:** Characteristics of the type of encounters between biopsy and definitive surgery.

Type of encounter	Mean or Median
	(Total *N* = 116 050)
*Number of new patient encounters in interval, any type*	
Mean (*SD*)	0.86 (0.95)
Median (Q1, Q3)	1.0 (0.0, 1.0)
*Number of unique medical oncologists consulted*	
Mean (*SD*)	0.16 (0.38)
Median (Q1, Q3)	0.0 (0.0, 0.0)
*Number of unique surgeons consulted*	
Mean (*SD*)	0.75 (0.56)
Median (Q1, Q3)	1.0 (0.0, 1.0)
*Number of unique radiation oncologists consulted*	
Mean (*SD*)	0.11 (0.32)
Median (Q1, Q3)	0.0 (0.0, 0.0)
*Number of established patient encounters in interval*	
Mean (*SD*)	1.36 (1.53)
Median (Q1, Q3)	1.0 (0.0, 2.0)
*Number of encounters in interval, any type*	
Mean (*SD*)	3.85 (3.35)
Median (Q1, Q3)	3.0 (2.0, 5.0)
*Number of biopsies in interval*	
Mean (*SD*)	0.16 (0.42)
Median (Q1, Q3)	0.0 (0.0, 0.0)
*Number of imaging studies in interval*	
Mean (*SD*)	0.48 (0.83)
Median (Q1, Q3)	0.0 (0.0, 1.0)
*Unique medical oncology, radiation oncology, or surgeon consultations as new patient visits*	
Mean (*SD*)	0.64 (0.80)
Median (Q1, Q3)	0.0 (0.0, 1.0)

*Note*: Q1, Q3 = Interquartile range.

Abbreviation: SD, standard deviation.

**TABLE 3:** Estimates of the subdistribution hazard ratios (sHR).

Number of evaluation components on different days	Sample Size	sHR at start of interval (i.e., main effect) sHR (95% CI)	Change in delay effect per month (i.e., interaction) sHR (95% CI)	Day after biopsy when effect of extra consultations equals that of having zero consultations Day (95% CI)
*New patient encounters* (*any specialty*)				
0 (Zero)	49 213	Reference = 1	0.99 (0.92, 1.06)	Reference
1	43 138	0.97 (0.89, 1.05)	1.02 (0.96, 1.09)	33.0 (−23.8, 89.7)
2	17 114	0.87 (0.76, 1.00)	1.12 (1.03, 1.22)	33.1 (13.5, 52.7)
3	4947	0.71 (0.55, 0.92)	1.36 (1.20, 1.55)	32.5 (16.5, 48.5)
4+	1638	0.63 (0.37, 1.07)	1.36 (1.13, 1.64)	43.5 (12.3, 74.7)
*Unique medical oncologists consulted* (*new and established visits*)				
0	98 664	Reference = 1	0.93 (0.89, 0.97)	Reference
1	16 791	0.81 (0.72, 0.91)	1.16 (1.08, 1.25)	29.3 (20.0, 38.6)
2+	595	0.62 (0.39, 1.00)	1.35 (1.13, 1.61)	38.1 (11.6, 64.6)
*Unique surgeons consulted* (*new and established visits*)				
	35 780	Reference = 1	0.96 (0.89, 1.03)	Reference
1	73 745	0.88 (0.81, 0.95)	1.10 (1.04, 1.15)	29.3 (17.3, 41.2)
2	6016	0.84 (0.68, 1.02)	1.13 (1.00, 1.27)	33.1 (11.6, 54.7)
3+	509	0.74 (0.39, 1.41)	1.00 (0.76, 1.31)	208 (−834, 1250)
*Unique radiation oncologists consulted* (*new and established visits*)				
0	103 888	Reference = 1	1.05 (1.00, 1.09)	Reference
1	11 948	0.87 (0.72, 1.05)	1.14 (0.99, 1.31)	49.1 (8.4, 89.7)
2+	214	1.11 (0.24, 5.17)	0.99 (0.51, 1.96)	63 (−492.3, 618.2)
*Established patient encounters*				
0	36 969	Reference = 1	0.90 (0.80, 1.00)	Reference
1	38 779	0.97 (0.88, 1.07)	0.94 (0.87, 1.03)	17.7 (−12.6, 48.1)
2	21 555	0.80 (0.70, 0.91)	1.11 (1.01, 1.23)	31.6 (19.8, 43.3)
3	9861	0.83 (0.68, 1.00)	1.08 (0.95, 1.22)	31.4 (13.1, 49.7)
4+	8886	0.70 (0.54, 0.89)	1.22 (1.12, 1.34)	35.1 (18.6, 51.5)
*Encounters of any type*				
0	7356	Reference = 1	0.65 (0.38, 1.11)	Reference
1	17 047	1.12 (0.89, 1.40)	0.70 (0.56, 0.87)	−52.2 (−612.4, 508.1)
2	21 794	1.03 (0.82, 1.29)	0.84 (0.71, 1.01)	−3.2 (−36.7, 30.3)
3	19 443	1.08 (0.86, 1.37)	0.77 (0.65, 0.90)	−15.4 (−110.1, 79.3)
4+	50 410	0.83 (0.67, 1.02)	1.10 (1.05, 1.16)	11.1 (4.0, 18.2)
*Biopsies*				
0	98 924	Reference = 1	1.06 (1.01, 1.10)	Reference
1	15 362	1.02 (0.91, 1.14)	1.03 (0.94, 1.12)	18.4 (−61.1, 98)
2+	1764	0.79 (0.53, 1.19)	1.05 (0.86, 1.29)	−1870 (−108 000, 104 000)
*Imaging studies*				
0	77 804	Reference = 1	0.91 (0.85, 0.96)	Reference
1	25 684	0.95 (0.86, 1.04)	1.04 (0.96, 1.12)	12.5 (−3.0, 27.9)
2	8791	1.03 (0.87, 1.22)	1.05 (0.94, 1.16)	−6.5 (−47.2, 34.2)
3+	3771	1.04 (0.70, 1.55)	1.12 (0.93, 1.35)	−5.6 (−67.6, 56.3)
*Unique medical oncologists, radiation oncologists, or surgeons consulted as new patient visits*				
0	60 933	Reference = 1	1.02 (0.96, 1.08)	Reference
1	39 887	0.93 (0.85, 1.01)	1.09 (1.02, 1.16)	37.7 (7.7, 67.8)
2	11 649	0.76 (0.64, 0.90)	1.24 (1.12, 1.38)	43.5 (27.6, 59.4)
3	3145	0.84 (0.55, 1.29)	1.38 (1.11, 1.73)	17.3 (−16.9, 51.5)
4+	436	0.36 (0.12, 1.07)	1.54 (1.04, 2.28)	76.6 (32.1, 121.1)

*Note*: Delay is defined as the interval between biopsy to definitive surgical treatment. The components count the number of days having the encounter or procedure of interest. We did not double count visits of the same type on the same day.

In Table 3, the effect of a one‐month delay is associated with increased mortality of sHR = 1.12 (95% CI 1.03–1.22) among those with two new patient encounters, sHR = 1.36 (CI 1.20–1.55) for those with three new patient encounters, and sHR = 1.36 (CI 1.13–1.64) for those with four or more. Interaction terms suggest that the *p*‐values for comparison of the sHRs for >1 new patient visits relative to having zero new patient visits were statistically significant (*p* < .03 for all three sHRs relative to the reference).

Increasing number of encounters seems to have a beneficial association in the short term before becoming detrimental with longer delays. The last column in Table 3 shows the day at which the sHR for number of new encounters greater than 0 crosses the black line representing the delay‐month specific risk of breast cancer mortality when a patient has 0 new encounters (33 days CI 14–53 for 2 new patient visits, 33 days CI 17–49 for 3 new patient visits, and 44 days CI 12–75 for 4+ new patient visits). The point estimates have a possible dose response benefit in the short term (sHR = 0.87 CI 0.76–1.00 for 2 new patient encounters, 0.71 CI 0.55–0.92 for 3, and 0.63 CI 0.37–1.07 for 4+ at minimal delay intercept).

**FIGURE 2:**
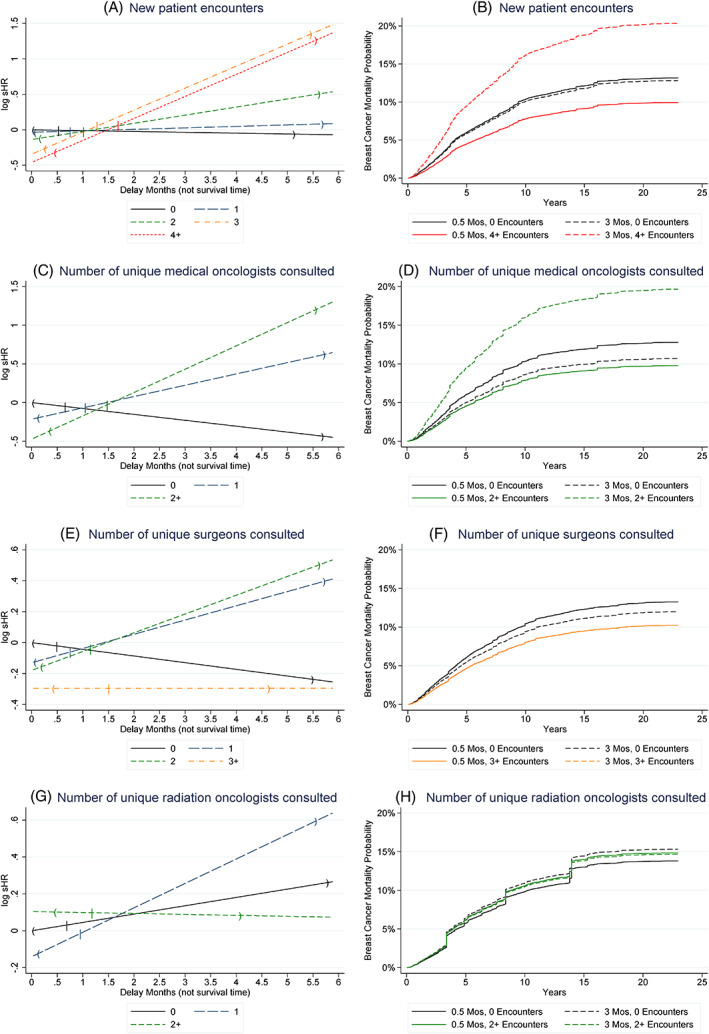
(A) Log subdistribution hazard ratios (sHRs) showing the effect of delay (as defined as time from biopsy to surgery) on breast cancer mortality by the number of new patient visits. Note that the reference category is the group having 0 new patient visits in the interval between biopsy and surgery (sHR = 1, log sHR = 0). The median is shown by | and extreme values beyond which <12 cases have delays are represented by parentheses. In the short term, increasing number of encounters is associated with better survival, but the estimates stop becoming protective relative to no delay and no extra encounters once the lines cross zero. (B) Representative cumulative incidence curves using estimates from the propensity score weighted competing risk regression for four groups defined by when the delay equals 0.5 or 3 months, and the number of new patient visits equals 0 or 4+. The curves demonstrate that for patients having no new patient encounters, there is minimal difference in the time between diagnosis and surgery, while those having 4 or more encounters note a large disparity in mortality when delay increases. This suggests that the impact of delay is incrementally more significant in those visiting more providers for the first time (i.e., having more new patient encounters). (C) Delay effects by number of unique medical oncologists consulted. (D) Representative cumulative incidence curves by number of unique medical oncologists consulted. (E) Delay effects by number of unique surgeons consulted. (F) Representative cumulative incidence curves by number of unique surgeons consulted. (G) Delay effects by number of unique radiation oncologists consulted. (H) Representative cumulative incidence curves by number of unique radiation oncologists consulted.

Figure 2 displays the time varying hazard ratios for each delay interval for salient findings. Figure 2A presents the log sHRs depicting the associations of new patient visits (0, 1, 2, 3, 4+ visits) by delay time with breast cancer specific mortality outcomes. In the figure, delay did not have much of an effect on outcomes in those with zero or one new patient visits in the biopsy to treatment interval. However, increasing time to surgery did seem to worsen outcomes for those with two or more new patient visits. The sHRs suggest that the direction of the effect of additional visits switches from a beneficial relationship (sHR < 1 and log sHR < 0) to a detrimental relationship (sHR > 1 and log sHR > 0) when the log sHRs cross the 0 y‐axis in Figure 2 (left column of figures). The effect of additional visits relative to 0 visits is equal when the lines intersect the black line that represents the delay‐dependent effect of having 0 visits. Figure 2B depicts estimated breast cancer specific cumulative incidence curves. As we measured delay on a continuous scale, we present representative adjusted cumulative incidence curves when the delay time is set to 0.5 or 3 months, or the number of encounters is set to 0 or the maximum category.

Physician specialty makes a difference in the relationship of clinical encounters with delay‐associated outcomes (Table 3 and Figure 2C,D). Encounters with two or more medical oncologists quickly are associated with reduced breast cancer specific mortality (sHR 0.81 CI 0.72–0.91 for 1 medical oncologist, sHR 0.62 CI 0.39–1.00 for two or more). A one‐month delay multiplies the sHR by 0.93 (CI 0.89–0.97), 1.16 (CI 1.08–1.25), and 1.35 (CI 1.13–1.61) for 0, 1, and 2+ medical oncologists, respectively. After 38 days (CI 12–65 days), the effect of having multiple medical oncologist visits begins to be associated with worsened cancer specific survival relative to having no medical oncologist visits. Consulting unique surgeons (Table 3 and Figure 2E,F) is also associated with beneficial breast cancer specific outcomes with baseline survival benefits of sHR 0.88 (CI 0.81–0.95), 0.84 (CI 0.68–1.02) and 0.74 (CI 0.39–1.41) for 1, 2 and 3+ unique surgeon visits. The reduction in the benefit associated with surgeon visits compared to no surgeon visits is eliminated at 29 days (CI 17–41) for 1 surgeon, but seems to persist beyond 6 months for 3+ surgeons (208 days until sHR for orange line representing 3+ surgeon encounters equals the black line relative to 0 surgeon visits, CI −834 to 1250 days for 3+ surgeons). Visiting multiple radiation oncologists does not have an impact (Table 3 and Figure 2G,H).

In Table 3 and supplemental Figures 1–9 for breast cancer specific mortality, having an increase in the number of established patient encounters was associated with improved breast cancer specific survival with 2 visits (sHR 0.80 CI 0.70–0.91 for 2 visits), with the benefit dissipating by 32 days (CI 20–43). Having 4 or more established patient visits was similarly associated with better baseline survival that declined over time (sHR = 0.70 CI 0.54–0.89 for 4+ established patient visits at baseline, with a relative monthly mortality increase of sHR = 1.22 CI 1.12–1.34 per month). The total number of encounters, which includes all visits within the interval, did not have a meaningful association with survival. Having multiple biopsies or images was not found to be related to cancer specific survival. The total number of unique physician consultations as new office visits, regardless of specialty, had inconsistent associations with survival.

For comparison, we present results for overall survival in supplemental Figures 1–9 and the associated Supplemental Tables. Boxplots depicting the distribution of delays by encounter groupings are also displayed in the Supplemental Figures.

## 4 DISCUSSION

Preoperative disease evaluation is beneficial for optimizing treatment, but too many preoperative visits can be associated with treatment delays that affect survival outcomes.^8^ This study attempts to delineate the extent to which delay reduces, and even reverses, the benefit of clinical evaluations before surgery. We found associations that may signify a trade‐off in delaying breast cancer treatment. Having multiple physicians evaluate patients in a short time seems to be associated with better outcomes. As the delay increases with multiple evaluations, the association of those evaluations with survival turns negative.

Second opinions may be one reason why many cases have numerous unique physician encounters. Morrow et al. found that 19.1% of surveyed women with breast cancer had a second opinion,^18^ and a recent study noted a 36% increase in transfers of care for breast cancer patients to another provider between 2004 and 2015.^5^ Having a higher education and having genetic variants of uncertain significance has been associated with seeking second opinions.^19^ While some studies have examined the reasons that patients seek second opinions and the resulting treatment decisions,^20^ there do not seem to be many studies examining second opinions' effect on survival.

Imaging for staging and identification of metastatic disease may also contribute to treatment delays.^21^ With greater survival and number of approved treatments for metastatic disease, it may not be surprising that imaging for breast cancer has increased over time.^22^ We did not find associations indicative of a survival impact of having repeated images or biopsies in the pre‐surgery interval.

While the benefit of additional evaluations and imaging encounters on survival is unclear, there is increasing evidence that treatment delays are associated with worse outcomes. A number of large studies have found that treatment delays are associated with worsened mortality.^3^, ^8^, ^23^ The effect of delay on outcomes may be causally related to tumor doubling times and rates of metastatic develpoment.^12^ Our findings provide evidence of the value of additional evaluations, where differences in treatment strategies have the potential to change a patient's outcome.

Although some consultations are necessary, and cannot be avoided because of the need to undergo treatment related to those providers, such as with a breast surgeon, plastic surgeon, or cardiologist, the value of having numerous opinions within the same specialty, or those not critical to the cancer workup preoperatively, may be questionable. This is not to say that multiple medical evaluations do not have value, but that the benefit of additional visits should be weighed against potential delays in cancer treatment that could impact survival.

Not all studies have found an effect of delays on survival, particularly in single institution studies. Li et al., for example, did not find an effect of treatment postponement of 90 days or more in a retrospective chart review.^24^ Similarly, Yoo et al. did not find an effect at Seoul National Hospital.^25^ Such single institution studies are limited by small sample sizes and loss to follow‐up if care is transferred. In Yoo et al.'s study, for example, only 1.8% had delays over 60 days. With a similarly small sample size, Wagner et al. did not find a statistically significant association between delay and tumor size progression among patients.^26^ However, the magnitude of the odds ratio describing the monthly effect of delay on predicting positive lymph node status was clinically relevant (OR = 1.31, 95% CI 0.95–1.82). Hence, literature concerning delay effects has to be considered with respect to the consistency of the size of estimated effects, even when not statistically significant.

Because the number of patients experiencing substantial delays is small, large population based registry studies like ours have greater power to demonstrate statistical significance. Ho et al. for example, found that waits greater than 90 days were associated with worse survival using Singapore registry data.^27^

One concern is that many studies do not sufficiently examine pathways by which delay impacts breast cancer survival outcomes. Comorbidities could cause postponement of surgery due to worsening clinical conditions not related to a primary breast cancer diagnosis. These co‐occurring diseases may require clinical management before surgery can be performed. This may be a particular concern in US Medicare samples over the age of 65. Delays could also be caused by rapid progression of disease which necessitates more intensive encounters prior to surgery. However, we excluded distant metastatic cases, likely limiting that impact here. Medicare claims do not provide reliable measures of changes in severity of disease that would allow for assessment of these effects.

Other limitations of this work are the potential for unknown unmeasured confounders to influence the results and the retrospective nature of linked SEER‐Medicare data. Also, the absolute reduction in mortality for some of our statistically significant findings might not be large. That is, statistical significance does not necessarily guarantee that findings are clinically relevant. Further, a substantial proportion were excluded due to missing biopsy or surgery date. While many of those might not have had definitive surgery as their first treatment,^28^, ^29^ a number who did have surgery were likely excluded due to missing data. Hence, it is possible that our findings might be biased due to missing data. In addition, immortal time bias may also be impacting our results since those with longer time to surgery necessarily must live long enough to have such a delay. However, the fact that longer wait times to surgery were generally associated with poorer outcomes, even when accounting for types of visits during the preoperative interval, suggests that any immortal time bias effect would be modest. Finally, the results are only generalizable to an older American population as those younger than 65 are not covered by Medicare or any other universal insurance scheme.

## 5 CONCLUSION

While treatment delays are generally associated with worse outcomes, we found that some postponement of surgery related to patient encounters with health care providers could be beneficial. For example, new patient visits, and particularly new medical oncology or surgery encounters, were associated with better survival outcomes if the encounters occurred within the first month after diagnosis. However, when accompanied by longer times to surgery, new patient visits were associated with worsened breast cancer mortality. Future research is needed to better understand the underlying reasons for these findings. For example, more detailed examination of those who have new patient encounters within the first month of diagnosis can identify care patterns that might be providing benefits to patients. Researchers could also examine possible mechanisms, such as disease progression, that might explain why encounters that occur with surgical delays of greater than 1 month are associated with worse mortality. Such future research will help clinicians assess when additional preoperative evaluations might be helpful, and when they might be harmful for patients.

## AUTHOR CONTRIBUTIONS

Brian L. Egleston: conceptualization, data curation, formal analysis, funding acquisition, investigation, methodology, project administration, visualization, writing – original draft. Richard J. Bleicher: conceptualization, data curation, funding acquisition, investigation, methodology, project administration, visualization, writing – original draft. Carolyn Y. Fang: conceptualization, funding acquisition, supervision, writing – original draft. Thomas J. Galloway: conceptualization, data curation, funding acquisition, investigation, methodology, writing – original draft. Slobodan Vucetic: conceptualization, data curation, formal analysis, funding acquisition, investigation, methodology, project administration, software, supervision, visualization, writing – original draft.

## FUNDING INFORMATION

This was funded in part by NIH/NCI grants R21CA202130 (PIs Egleston/Vucetic), U54CA221705, and P30CA006927 (Fox Chase Cancer Center Support Grant).

## CONFLICT OF INTEREST STATEMENT

The authors have stated explicitly that there are no conflicts of interest in connection with this article.

## ETHICS STATEMENT

The Fox Chase Cancer Center Institutional Review Board approved the project, determining that the study is exempt (US 45 CFR 46.110(b) (1) exempt category 4, IRB ID 17‐9001).

## PRECIS

Additional evaluations before breast cancer surgery may improve care, but may cause detrimental delays. We investigate when surgical delays may be helpful by allowing additional clinical visits that optimize care, versus when delays may be harmful due to the potential for disease progression. We use population‐based SEER‐Medicare registry data.

## Supporting information


**Data S1:** Supporting Information.

## Data Availability

The SEER‐Medicare data that support the findings of this study are available from the U.S. National Cancer Institute.
