# Supplementary material for: Benefits versus drawbacks of delaying surgery due to additional consultations in older patients with breast cancer
Source: Cancer Rep (Hoboken). 2023 Mar 21;6(5):e1805. doi: 10.1002/cnr2.1805 (PMC10172180; doi:10.1002/cnr2.1805)
Supplement: Supplementary file 1 — Data S1: Supporting Information. [file CNR2-6-e1805-s001.docx]

**Table of Contents: Supplemental Material for “Benefits versus drawbacks of delaying surgery due to additional consultations in older patients with breast cancer”**

**Pages 3-8. Supplemental Tables 1-4: New Patient Encounters, summary characteristics. “***” used to hide numbers <12 for privacy.**

Supplemental Table 1: New Patient Encounters, continuous demographic and clinical variables.

Supplemental Table 2: New Patient Encounters, categorical demographic and clinical variables.

Supplemental Table 3: New Patient Encounters, continuous demographic and clinic al variables after propensity score based weighting.

Supplemental Table 4: New Patient Encounters, categorical demographic and clinical variables after propensity score based weighting.

**Pages 9-13. Supplemental Tables 5-8: Number of unique medical oncologists consulted, summary characteristics. “***” used to hide numbers <12 for privacy.**

Supplemental Table 5: Number of unique medical oncologists consulted, continuous demographic and clinical variables.

Supplemental Table 6: Number of unique medical oncologists consulted, categorical demographic and clinical variables.

Supplemental Table 7: Number of unique medical oncologists consulted, continuous demographic and clinical variables after propensity score based weighting.

Supplemental Table 8: Number of unique medical oncologists consulted, categorical demographic and clinical variables after propensity score based weighting.

**Pages 14-19. Supplemental Tables 9-12: Number of unique surgeons consulted, summary characteristics. “***” used to hide numbers <12 for privacy.**

Supplemental Table 9: Number of unique surgeons consulted, continuous demographic and clinical variables.

Supplemental Table 10: Number of unique surgeons consulted, categorical demographic and clinical variables.

Supplemental Table 11: Number of unique surgeons consulted, continuous demographic and clinical variables after propensity score based weighting.

Supplemental Table 12: Number of unique surgeons consulted, categorical demographic and clinical variables after propensity score based weighting.

**Page 20. Supplemental Figure 1: New Patient Encounters.** A. Breast Cancer Specific Mortality Estimates Using Competing Risk Regression Results. B. Subdistribution Hazard Ratios from Breast Cancer Specific Competing Risk Regressions. Extreme delay interval values beyond which there are fewer than 12 cases within each group are marked by parentheses. The median within the group is marked by a vertical bar. C. Boxplot Showing Distribution of Delay Interval in Days by New Patient Encounters. D. Overall Survival Estimates Using Cox Regression Results E. Overall Survival Hazard Ratios from Cox Regressions.

**Page 21. Supplemental Table 13.**  Estimates akin to those presented in Table 3 for: New Patient Encounters.

**Page 22. Supplemental Figure 2: Number of unique medical oncologists consulted.** A. Breast Cancer Specific Mortality Estimates Using Competing Risk Regression Results. B. Subdistribution Hazard Ratios from Breast Cancer Specific Competing Risk Regressions. Extreme delay interval values beyond which there are fewer than 12 cases within each group are marked by parentheses. The median within the group is marked by a vertical bar. C. Boxplot Showing Distribution of Delay Interval in Days by New Patient Encounters. D. Overall Survival Estimates Using Cox Regression Results E. Overall Survival Hazard Ratios from Cox Regressions.

**Page 23. Supplemental Table 14.** Estimates akin to those presented in Table 3 for number of unique medical oncologists consulted.

**Page 24. Supplemental Figure 3: Number of surgeons consulted.** A. Breast Cancer Specific Mortality Estimates Using Competing Risk Regression Results. B. Subdistribution Hazard Ratios from Breast Cancer Specific Competing Risk Regressions. Extreme delay interval values beyond which there are fewer than 12 cases within each group are marked by parentheses. The median within the group is marked by a vertical bar. C. Boxplot Showing Distribution of Delay Interval in Days by New Patient Encounters. D. Overall Survival Estimates Using Cox Regression Results E. Overall Survival Hazard Ratios from Cox Regressions.

**Page 25. Supplemental Table 15.** Estimates akin to those presented in Table 3 for number of unique surgeons consulted.

**Page 26. Supplemental Figure 4: Number of unique radiation oncologists consulted.** A. Breast Cancer Specific Mortality Estimates Using Competing Risk Regression Results. B. Subdistribution Hazard Ratios from Breast Cancer Specific Competing Risk Regressions. Extreme delay interval values beyond which there are fewer than 12 cases within each group are marked by parentheses. The median within the group is marked by a vertical bar. C. Boxplot Showing Distribution of Delay Interval in Days by New Patient Encounters. D. Overall Survival Estimates Using Cox Regression Results E. Overall Survival Hazard Ratios from Cox Regressions.

**Page 27. Supplemental Table 16.** Estimates akin to those presented in Table 3 for number of unique radiation oncologists consulted.

**Page 28. Supplemental Figure 5: Established physician encounters.** A. Breast Cancer Specific Mortality Estimates Using Competing Risk Regression Results. B. Subdistribution Hazard Ratios from Breast Cancer Specific Competing Risk Regressions. Extreme delay interval values beyond which there are fewer than 12 cases within each group are marked by parentheses. The median within the group is marked by a vertical bar. C. Boxplot Showing Distribution of Delay Interval in Days by New Patient Encounters. D. Overall Survival Estimates Using Cox Regression Results E. Overall Survival Hazard Ratios from Cox Regressions.

**Page 29. Supplemental Table 17.** Estimates akin to those presented in Table 3 for established patient encounters.

**Page 30. Supplemental Figure 6: Number of encounters.** A. Breast Cancer Specific Mortality Estimates Using Competing Risk Regression Results. B. Subdistribution Hazard Ratios from Breast Cancer Specific Competing Risk Regressions. Extreme delay interval values beyond which there are fewer than 12 cases within each group are marked by parentheses. The median within the group is marked by a vertical bar. C. Boxplot Showing Distribution of Delay Interval in Days by New Patient Encounters. D. Overall Survival Estimates Using Cox Regression Results E. Overall Survival Hazard Ratios from Cox Regressions.

**Page 31. Supplemental Table 18.** Estimates akin to those presented in Table 3 for number of encounters any type.

**Page 32. Supplemental Figure 7: Number of biopsies.** A. Breast Cancer Specific Mortality Estimates Using Competing Risk Regression Results. B. Subdistribution Hazard Ratios from Breast Cancer Specific Competing Risk Regressions. Extreme delay interval values beyond which there are fewer than 12 cases within each group are marked by parentheses. The median within the group is marked by a vertical bar. C. Boxplot Showing Distribution of Delay Interval in Days by New Patient Encounters. D. Overall Survival Estimates Using Cox Regression Results E. Overall Survival Hazard Ratios from Cox Regressions.

**Page 33. Supplemental Table 19.** Estimates akin to those presented in Table 3 for number of biopsies.

**Page 34. Supplemental Figure 8: Number of imaging studies.** A. Breast Cancer Specific Mortality Estimates Using Competing Risk Regression Results. B. Subdistribution Hazard Ratios from Breast Cancer Specific Competing Risk Regressions. Extreme delay interval values beyond which there are fewer than 12 cases within each group are marked by parentheses. The median within the group is marked by a vertical bar. C. Boxplot Showing Distribution of Delay Interval in Days by New Patient Encounters. D. Overall Survival Estimates Using Cox Regression Results E. Overall Survival Hazard Ratios from Cox Regressions.

**Page 35. Supplemental Table 20.** Estimates akin to those presented in Table 3 for number of imaging studies.

**Page 36. Supplemental Figure 9: Number of unique physicians consulted as new patient visits (medical oncologists, surgeons, or radiation oncologists only).** A. Breast Cancer Specific Mortality Estimates Using Competing Risk Regression Results. B. Subdistribution Hazard Ratios from Breast Cancer Specific Competing Risk Regressions. Extreme delay interval values beyond which there are fewer than 12 cases within each group are marked by parentheses. The median within the group is marked by a vertical bar. C. Boxplot Showing Distribution of Delay Interval in Days by New Patient Encounters. D. Overall Survival Estimates Using Cox Regression Results E. Overall Survival Hazard Ratios from Cox Regressions.

**Pages 37-38. Supplemental Table 21.** Estimates akin to those presented in Table 3 for unique physicians consulted as new patient visits (medical oncologists, surgeons, or radiation oncologists only).

**Page 39. Appendix.** SAS formats used to identify treatments and physician encounters.

Supplemental Table 1: New Patient Encounters, continuous demographic and clinical variables

| **Number of new patient encounters** | 0 | 0 | 0 | 1 | 1 | 1 | 2 | 2 | 2 | 3 | 3 | 3 | 4+ | 4+ | 4+ |  |
| --- | --- | --- | --- | --- | --- | --- | --- | --- | --- | --- | --- | --- | --- | --- | --- | --- |
|  | N | mean | SD | N | mean | SD | N | mean | SD | N | mean | SD | N | mean | SD | p-value |
| **Days from biopsy to surgery** | | | | | | | | | | | | | | | | |
|  | 49213 | 19.09 | 14.19 | 43138 | 27.28 | 17.58 | 17114 | 35.87 | 21.79 | 4947 | 45.27 | 26.29 | 1638 | 61.14 | 33.88 | 0.00000 |
| **Age at Diagnosis** | | | | | | | | | | | | | | | | |
|  | 49213 | 75.27 | 6.23 | 43138 | 75.01 | 6.16 | 17114 | 74.70 | 6.12 | 4947 | 74.27 | 6.03 | 1638 | 73.32 | 5.98 | 0.00000 |
| **% of Zip Code with Less than a High School Education** | | | | | | | | | | | | | | | | |
|  | 49213 | 16.90 | 10.62 | 43138 | 14.31 | 10.07 | 17114 | 13.56 | 10.01 | 4947 | 12.55 | 9.61 | 1638 | 12.20 | 9.73 | 0.00000 |
| **% of Zip Code Living Below Poverty Line** | | | | | | | | | | | | | | | | |
|  | 49213 | 12.72 | 8.71 | 43138 | 12.11 | 8.40 | 17114 | 11.80 | 8.32 | 4947 | 11.36 | 8.03 | 1638 | 11.37 | 8.44 | 0.00000 |
| **Charlson Comorbidity Index** | | | | | | | | | | | | | | | | |
|  | 49213 | 0.67 | 1.10 | 43138 | 0.63 | 1.05 | 17114 | 0.65 | 1.07 | 4947 | 0.66 | 1.08 | 1638 | 0.64 | 1.05 | 0.00000 |
| **Elixhauser Score** | | | | | | | | | | | | | | | | |
|  | 49213 | 2.12 | 5.54 | 43138 | 1.75 | 5.11 | 17114 | 1.74 | 5.11 | 4947 | 1.76 | 5.26 | 1638 | 1.44 | 4.54 | 0.00000 |
| **Sequence Number of Cancer** | | | | | | | | | | | | | | | | |
|  | 49213 | 1.08 | 0.30 | 43138 | 1.08 | 0.31 | 17114 | 1.08 | 0.30 | 4947 | 1.09 | 0.32 | 1638 | 1.07 | 0.29 | 0.05200 |
| **Tumor Size (mm)** | | | | | | | | | | | | | | | | |
|  | 49213 | 19.32 | 19.27 | 43138 | 18.22 | 19.74 | 17114 | 18.55 | 17.79 | 4947 | 18.99 | 15.31 | 1638 | 19.79 | 16.45 | 0.00000 |
| **Year of Breast Cancer Diagnosis** | | | | | | | | | | | | | | | | |
|  | 49213 | 2003.74 | 5.71 | 43138 | 2005.71 | 5.24 | 17114 | 2006.40 | 4.98 | 4947 | 2006.85 | 4.83 | 1638 | 2006.86 | 4.77 | 0.00000 |
| **Number of Lymph Nodes Positive** | | | | | | | | | | | | | | | | |
|  | 49213 | 0.93 | 2.78 | 43138 | 0.81 | 2.59 | 17114 | 0.83 | 2.70 | 4947 | 0.87 | 2.74 | 1638 | 1.04 | 3.00 | 0.00000 |

Supplemental Table 2: New Patient Encounters, categorical demographic and clinical variables

| **Number of new patient encounters** | 0 | 0 | 1 | 1 | 2 | 2 | 3 | 3 | 4+ | 4+ |  |
| --- | --- | --- | --- | --- | --- | --- | --- | --- | --- | --- | --- |
|  | N | % | N | % | N | % | N | % | N | % | p-value |
| **Interval (Days)** |  |  |  |  |  |  |  |  |  |  | 0.00000 |
| 1-30 | 41926 | 85.2 | 29912 | 69.3 | 8500 | 49.7 | 1613 | 32.6 | 258 | 15.8 |  |
| 31-60 | 6450 | 13.1 | 11265 | 26.1 | 6873 | 40.2 | 2337 | 47.2 | 752 | 45.9 |  |
| 61-90 | 639 | 1.3 | 1485 | 3.4 | 1256 | 7.3 | 664 | 13.4 | 335 | 20.5 |  |
| 91-120 | 127 | 0.3 | 301 | 0.7 | 304 | 1.8 | 213 | 4.3 | 171 | 10.4 |  |
| 121-180 | 71 | 0.1 | 175 | 0.4 | 181 | 1.1 | 120 | 2.4 | 122 | 7.4 |  |
| **Background** |  |  |  |  |  |  |  |  |  |  | 0.00000 |
| European Ancestry | 43483 | 88.4 | 38449 | 89.1 | 15105 | 88.3 | 4362 | 88.2 | 1452 | 88.6 |  |
| African Ancestry | 3506 | 7.1 | 2490 | 5.8 | 1092 | 6.4 | 324 | 6.5 | 107 | 6.5 |  |
| Other or Unknown | 789 | 1.6 | 845 | 2.0 | 341 | 2.0 | 92 | 1.9 | 34 | 2.1 |  |
| Asian | 969 | 2.0 | 930 | 2.2 | 376 | 2.2 | 104 | 2.1 | 29 | 1.8 |  |
| Hispanic | 466 | 0.9 | 424 | 1.0 | 200 | 1.2 | 65 | 1.3 | 16 | 1.0 |  |
| **Census Region of the United States** |  |  |  |  |  |  |  |  |  |  | 0.00000 |
| Northeast | 9665 | 19.6 | 7306 | 16.9 | 2932 | 17.1 | 855 | 17.3 | 301 | 18.4 |  |
| Midwest | 7516 | 15.3 | 6842 | 15.9 | 3171 | 18.5 | 856 | 17.3 | 226 | 13.8 |  |
| West | 17490 | 35.5 | 20867 | 48.4 | 8723 | 51.0 | 2756 | 55.7 | 962 | 58.7 |  |
| South | 14542 | 29.5 | 8123 | 18.8 | 2288 | 13.4 | 480 | 9.7 | 149 | 9.1 |  |
| **Marital Status** |  |  |  |  |  |  |  |  |  |  | 0.00000 |
| Not Married | 27075 | 55.0 | 22451 | 52.0 | 8938 | 52.2 | 2511 | 50.8 | 829 | 50.6 |  |
| Married | 22138 | 45.0 | 20687 | 48.0 | 8176 | 47.8 | 2436 | 49.2 | 809 | 49.4 |  |
| **Type of Metropolitan Area** |  |  |  |  |  |  |  |  |  |  | 0.00000 |
| Big Metro | 24625 | 50.0 | 23799 | 55.2 | 10447 | 61.0 | 3222 | 65.1 | 1143 | 69.8 |  |
| Metro | 14725 | 29.9 | 13229 | 30.7 | 4595 | 26.8 | 1196 | 24.2 | 364 | 22.2 |  |
| Urban | 3430 | 7.0 | 2183 | 5.1 | 778 | 4.5 | 238 | 4.8 | 56 | 3.4 |  |
| Less Urban/Rural | 6433 | 13.1 | 3927 | 9.1 | 1294 | 7.6 | 291 | 5.9 | 75 | 4.6 |  |
| **SEER Defined Sex** |  |  |  |  |  |  |  |  |  |  | 0.00000 |
| Male | 571 | 1.2 | 375 | 0.9 | 86 | 0.5 | *** | *** | *** | *** |  |
| Female | 48642 | 98.8 | 42763 | 99.1 | 17028 | 99.5 | *** | *** | *** | *** |  |
| Histology |  |  |  |  |  |  |  |  |  |  | 0.00000 |
| Ductal | 42463 | 86.3 | 37574 | 87.1 | 14787 | 86.4 | 4272 | 86.4 | 1389 | 84.8 |  |
| Lobular | 5437 | 11.0 | 4690 | 10.9 | 2016 | 11.8 | 589 | 11.9 | 221 | 13.5 |  |
| Other or Unknown | 1313 | 2.7 | 874 | 2.0 | 311 | 1.8 | 86 | 1.7 | 28 | 1.7 |  |
| **Grade** |  |  |  |  |  |  |  |  |  |  | 0.00000 |
| Well | 10971 | 22.3 | 10824 | 25.1 | 4248 | 24.8 | 1172 | 23.7 | 393 | 24.0 |  |
| Moderate | 20767 | 42.2 | 18901 | 43.8 | 7677 | 44.9 | 2280 | 46.1 | 759 | 46.3 |  |
| Poor | 12298 | 25.0 | 10361 | 24.0` | 4105 | 24.0 | 1201 | 24.3 | 388 | 23.7 |  |
| Undifferentiated | 582 | 1.2 | 335 | 0.8 | 141 | 0.8 | *** | *** | *** | *** |  |
| Unknown | 4595 | 9.3 | 2717 | 6.3 | 943 | 5.5 | *** | *** | *** | *** |  |
| **ER or PR Positive** |  |  |  |  |  |  |  |  |  |  | 0.00000 |
| Negative | 11532 | 23.4 | 8317 | 19.3 | 3159 | 18.5 | 871 | 17.6 | 301 | 18.4 |  |
| Positive | 37681 | 76.6 | 34821 | 80.7 | 13955 | 81.5 | 4076 | 82.4 | 1337 | 81.6 |  |
| **HER2 Positive** |  |  |  |  |  |  |  |  |  |  | 0.00000 |
| Negative | 48348 | 98.2 | 42041 | 97.5 | 16647 | 97.3 | 4786 | 96.7 | 1591 | 97.1 |  |
| Positive | 865 | 1.8 | 1097 | 2.5 | 467 | 2.7 | 161 | 3.3 | 47 | 2.9 |  |
| **AJCC Stage** |  |  |  |  |  |  |  |  |  |  | 0.00000 |
| Stage 1 | 28204 | 57.3 | 26318 | 61.0 | 10251 | 59.9 | 2863 | 57.9 | 892 | 54.5 |  |
| Stage 2 | 17851 | 36.3 | 14287 | 33.1 | 5777 | 33.8 | 1727 | 34.9 | 601 | 36.7 |  |
| Stage 3 | 3158 | 6.4 | 2533 | 5.9 | 1086 | 6.3 | 357 | 7.2 | 145 | 8.9 |  |
| Mastectomy |  |  |  |  |  |  |  |  |  |  | 0.00000 |
| Lumpectomy Only | 22560 | 45.8 | 24539 | 56.9 | 9674 | 56.5 | 2745 | 55.5 | 758 | 46.3 |  |
| Mastectomy | 26653 | 54.2 | 18599 | 43.1 | 7440 | 43.5 | 2202 | 44.5 | 880 | 53.7 |  |
| **Were nodes examined** |  |  |  |  |  |  |  |  |  |  | 0.00000 |
| No | 1039 | 2.1 | 632 | 1.5 | 247 | 1.4 | 76 | 1.5 | 35 | 2.1 |  |
| Yes | 48174 | 97.9 | 42506 | 98.5 | 16867 | 98.6 | 4871 | 98.5 | 1603 | 97.9 |  |
| **Reconstructive Surgery** |  |  |  |  |  |  |  |  |  |  | 0.00000 |
| No | 48860 | 99.3 | 41908 | 97.1 | 15840 | 92.6 | 4345 | 87.8 | 1314 | 80.2 |  |
| Yes | 353 | 0.7 | 1230 | 2.9 | 1274 | 7.4 | 602 | 12.2 | 324 | 19.8 |  |
| **Adjuvant Chemotherapy** |  |  |  |  |  |  |  |  |  |  | 0.00000 |
| No Adjuvant | 37779 | 76.8 | 33063 | 76.6 | 12792 | 74.7 | 3679 | 74.4 | 1193 | 72.8 |  |
| Adjuvant | 11434 | 23.2 | 10075 | 23.4 | 4322 | 25.3 | 1268 | 25.6 | 445 | 27.2 |  |
| **Adjuvant Radiotherapy** |  |  |  |  |  |  |  |  |  |  | 0.00000 |
| No Radiotherapy Adjuvant | 25200 | 51.2 | 18151 | 42.1 | 7091 | 41.4 | 2075 | 41.9 | 793 | 48.4 |  |
| Radiotherapy Adjuvant | 24013 | 48.8 | 24987 | 57.9 | 10023 | 58.6 | 2872 | 58.1 | 845 | 51.6 |  |

***** used to hide numbers <12 for privacy.**

Supplemental Table 3: New Patient Encounters, continuous demographic and clinical variables after propensity score based weighting

| **Number of new patient encounters** | 0 | 0 | 0 | 1 | 1 | 1 | 2 | 2 | 2 | 3 | 3 | 3 | 4+ | 4+ | 4+ |
| --- | --- | --- | --- | --- | --- | --- | --- | --- | --- | --- | --- | --- | --- | --- | --- |
|  | N | mean | SD | N | mean | SD | N | mean | SD | N | mean | SD | N | Mean | SD |
| **Days from biopsy to surgery (not in model)** | | | | | | | | | | | | | | | |
|  | 49213 | 20.01 | 14.62 | 43138 | 27.14 | 17.74 | 17114 | 35.08 | 22.00 | 4947 | 45.07 | 27.56 | 1638 | 60.39 | 33.41 |
| **Age at Diagnosis** | | | | | | | | | | | | | | | |
|  | 49213 | 75.03 | 6.18 | 43138 | 75.02 | 6.18 | 17114 | 74.98 | 6.19 | 4947 | 75.01 | 6.23 | 1638 | 74.96 | 6.14 |
| **% of Zip Code with Less than a High School Education** | | | | | | | | | | | | | | | |
|  | 49213 | 15.15 | 10.35 | 43138 | 15.23 | 10.43 | 17114 | 15.11 | 10.30 | 4947 | 15.52 | 10.59 | 1638 | 16.27 | 11.29 |
| **% of Zip Code Living Below Poverty Line** | | | | | | | | | | | | | | | |
|  | 49213 | 12.22 | 8.45 | 43138 | 12.26 | 8.57 | 17114 | 12.17 | 8.51 | 4947 | 12.33 | 8.67 | 1638 | 13.15 | 9.35 |
| **Charlson Comorbidity Index** | | | | | | | | | | | | | | | |
|  | 49213 | 0.65 | 1.07 | 43138 | 0.65 | 1.08 | 17114 | 0.64 | 1.06 | 4947 | 0.65 | 1.06 | 1638 | 0.70 | 1.12 |
| **Elixhauser Score** |  |  |  |  |  |  |  |  |  |  |  |  |  |  |  |
|  | 49213 | 1.92 | 5.32 | 43138 | 1.90 | 5.30 | 17114 | 1.92 | 5.31 | 4947 | 1.90 | 5.29 | 1638 | 2.12 | 5.43 |
| **Sequence Number of Cancer** | | | | | | | | | | | | | | | |
|  | 49213 | 1.08 | 0.31 | 43138 | 1.08 | 0.30 | 17114 | 1.08 | 0.30 | 4947 | 1.08 | 0.29 | 1638 | 1.08 | 0.32 |
| **Tumor Size** |  |  |  |  |  |  |  |  |  |  |  |  |  |  |  |
|  | 49213 | 18.92 | 19.37 | 43138 | 18.87 | 19.17 | 17114 | 18.92 | 18.50 | 4947 | 18.74 | 15.04 | 1638 | 19.41 | 18.59 |
| **Year of Breast Cancer Diagnosis** | | | | | | | | | | | | | | | |
|  | 49213 | 2005.01 | 5.50 | 43138 | 2004.99 | 5.53 | 17114 | 2004.94 | 5.59 | 4947 | 2004.77 | 5.76 | 1638 | 2004.72 | 5.70 |
| **Number of Lymph Nodes Positive** | | | | | | | | | | | | | | | |
|  | 49213 | 0.87 | 2.70 | 43138 | 0.87 | 2.70 | 17114 | 0.87 | 2.67 | 4947 | 0.94 | 2.79 | 1638 | 0.82 | 2.53 |

Supplemental Table 4: New Patient Encounters, categorical demographic and clinical variables after propensity score based weighting

| **Number of new patient encounters** | 0 | 0 | 1 | 1 | 2 | 2 | 3 | 3 | 4+ | 4+ |
| --- | --- | --- | --- | --- | --- | --- | --- | --- | --- | --- |
|  | N | % | N | % | N | % | N | % | N | % |
| **Interval (Days, not in model)** |  |  |  |  |  |  |  |  |  |  |
| 1-30 | 41926 | 83.4 | 29912 | 69.7 | 8500 | 52.2 | 1613 | 34.9 | 258 | 17.2 |
| 31-60 | 6450 | 14.7 | 11265 | 25.7 | 6873 | 38.1 | 2337 | 45.0 | 752 | 44.9 |
| 61-90 | 639 | 1.5 | 1485 | 3.4 | 1256 | 6.8 | 664 | 12.8 | 335 | 20.2 |
| 91-120 | 127 | 0.3 | 301 | 0.7 | 304 | 1.7 | 213 | 4.5 | 171 | 11.0 |
| 121-180 | 71 | 0.1 | 175 | 0.4 | 181 | 1.1 | 120 | 2.8 | 122 | 6.7 |
| **Background** |  |  |  |  |  |  |  |  |  |  |
| European Ancestry | 43483 | 88.7 | 38449 | 88.6 | 15105 | 88.5 | 4362 | 88.4 | 1452 | 86.6 |
| African Ancestry | 3506 | 6.4 | 2490 | 6.5 | 1092 | 6.6 | 324 | 7.2 | 107 | 8.6 |
| Other or Unknown | 789 | 1.8 | 845 | 1.8 | 341 | 1.8 | 92 | 1.6 | 34 | 1.6 |
| Asian | 969 | 2.0 | 930 | 2.1 | 376 | 2.1 | 104 | 1.9 | 29 | 2.2 |
| Hispanic | 466 | 1.0 | 424 | 1.0 | 200 | 1.0 | 65 | 0.9 | 16 | 1.0 |
| **Census Region of the United States** |  |  |  |  |  |  |  |  |  |  |
| Northeast | 9665 | 18.9 | 7306 | 18.5 | 2932 | 19.1 | 855 | 20.0 | 301 | 18.3 |
| Midwest | 7516 | 16.2 | 6842 | 16.2 | 3171 | 15.9 | 856 | 16.2 | 226 | 17.8 |
| West | 17490 | 43.1 | 20867 | 43.3 | 8723 | 43.3 | 2756 | 41.6 | 962 | 40.8 |
| South | 14542 | 21.8 | 8123 | 22.0 | 2288 | 21.8 | 480 | 22.3 | 149 | 23.2 |
| **Marital Status** |  |  |  |  |  |  |  |  |  |  |
| Not married | 27075 | 53.5 | 22451 | 53.3 | 8938 | 53.2 | 2511 | 53.6 | 829 | 52.9 |
| Married | 22138 | 46.5 | 20687 | 46.7 | 8176 | 46.8 | 2436 | 46.4 | 809 | 47.1 |
| **Type of Metropolitan Area** |  |  |  |  |  |  |  |  |  |  |
| Big Metro | 24625 | 54.4 | 23799 | 54.6 | 10447 | 54.5 | 3222 | 52.5 | 1143 | 52.1 |
| Metro | 14725 | 29.3 | 13229 | 29.2 | 4595 | 29.4 | 1196 | 30.6 | 364 | 29.0 |
| Urban | 3430 | 5.8 | 2183 | 5.9 | 778 | 5.8 | 238 | 5.8 | 56 | 6.3 |
| Less Urban/Rural | 6433 | 10.5 | 3927 | 10.4 | 1294 | 10.2 | 291 | 11.1 | 75 | 12.6 |
| **SEER Defined Sex** |  |  |  |  |  |  |  |  |  |  |
| Male | 571 | 0.9 | 375 | 1.0 | 86 | 1.0 | *** | *** | *** | *** |
| Female | 48642 | 99.1 | 42763 | 99.0 | 17028 | 99.0 | *** | *** | *** | *** |
| **Histology** |  |  |  |  |  |  |  |  |  |  |
| Ductal | 42463 | 86.6 | 37574 | 86.6 | 14787 | 86.6 | 4272 | 86.2 | 1389 | 85.8 |
| Lobular | 5437 | 11.1 | 4690 | 11.1 | 2016 | 11.2 | 589 | 11.4 | 221 | 11.6 |
| Other or Unknown | 1313 | 2.3 | 874 | 2.3 | 311 | 2.2 | 86 | 2.4 | 28 | 2.6 |
| **Grade** |  |  |  |  |  |  |  |  |  |  |
| Well | 10971 | 23.8 | 10824 | 23.7 | 4248 | 23.5 | 1172 | 22.8 | 393 | 23.1 |
| Moderate | 20767 | 43.2 | 18901 | 43.3 | 7677 | 43.3 | 2280 | 44.0 | 759 | 42.2 |
| Poor | 12298 | 24.5 | 10361 | 24.4 | 4105 | 24.8 | 1201 | 23.4 | 388 | 25.3 |
| Undifferentiated | 582 | 1.0 | 335 | 1.0 | 141 | 1.0 | *** | *** | *** | *** |
| Unknown | 4595 | 7.5 | 2717 | 7.6 | 943 | 7.5 | *** | *** | *** | *** |
| **ER or PR Positive** |  |  |  |  |  |  |  |  |  |  |
| Negative | 11532 | 20.9 | 8317 | 21.0 | 3159 | 21.3 | 871 | 21.9 | 301 | 24.0 |
| Positive | 37681 | 79.1 | 34821 | 79.0 | 13955 | 78.7 | 4076 | 78.1 | 1337 | 76.0 |
| **HER2 Positive** |  |  |  |  |  |  |  |  |  |  |
| Negative | 48348 | 97.7 | 42041 | 97.7 | 16647 | 97.7 | 4786 | 98.0 | 1591 | 98.2 |
| Positive | 865 | 2.3 | 1097 | 2.3 | 467 | 2.3 | 161 | 2.0 | 47 | 1.8 |
| **AJCC Stage** |  |  |  |  |  |  |  |  |  |  |
| Stage 1 | 28204 | 58.7 | 26318 | 58.9 | 10251 | 58.5 | 2863 | 58.3 | 892 | 58.2 |
| Stage 2 | 17851 | 34.9 | 14287 | 34.8 | 5777 | 35.2 | 1727 | 35.1 | 601 | 35.2 |
| Stage 3 | 3158 | 6.4 | 2533 | 6.4 | 1086 | 6.3 | 357 | 6.5 | 145 | 6.6 |
| **Mastectomy** |  |  |  |  |  |  |  |  |  |  |
| Lumpectomy Only | 22560 | 52.2 | 24539 | 51.7 | 9674 | 52.0 | 2745 | 53.1 | 758 | 53.4 |
| Mastectomy | 26653 | 47.8 | 18599 | 48.3 | 7440 | 48.0 | 2202 | 46.9 | 880 | 46.6 |
| **Were nodes examined** |  |  |  |  |  |  |  |  |  |  |
| No | 1039 | 1.9 | 632 | 1.8 | 247 | 1.8 | 76 | 2.2 | 35 | 3.2 |
| Yes | 48174 | 98.1 | 42506 | 98.2 | 16867 | 98.2 | 4871 | 97.8 | 1603 | 96.8 |
| **Reconstructive Surgery** |  |  |  |  |  |  |  |  |  |  |
| No | 48860 | 96.0 | 41908 | 96.8 | 15840 | 96.7 | 4345 | 96.9 | 1314 | 96.9 |
| Yes | 353 | 4.0 | 1230 | 3.2 | 1274 | 3.3 | 602 | 3.1 | 324 | 3.1 |
| **Adjuvant Chemotherapy** |  |  |  |  |  |  |  |  |  |  |
| No Adjuvant | 37779 | 76.1 | 33063 | 76.2 | 12792 | 76.2 | 3679 | 76.1 | 1193 | 75.5 |
| Adjuvant | 11434 | 23.9 | 10075 | 23.8 | 4322 | 23.8 | 1268 | 23.9 | 445 | 24.5 |
| **Adjuvant Radiotherapy** |  |  |  |  |  |  |  |  |  |  |
| No Radiotherapy Adjuvant | 25200 | 45.4 | 18151 | 46.0 | 7091 | 45.6 | 2075 | 44.3 | 793 | 43.4 |
| Radiotherapy Adjuvant | 24013 | 54.6 | 24987 | 54.0 | 10023 | 54.4 | 2872 | 55.7 | 845 | 56.6 |

***** used to hide numbers <12 for privacy.**

Supplemental Table 5: Number of unique medical oncologists consulted, continuous demographic and clinical variables

| **Number of unique medical oncologists consulted** | 0 | 0 | 0 | 1 | 1 | 1 | 2+ | 2+ | 2+ |  |
| --- | --- | --- | --- | --- | --- | --- | --- | --- | --- | --- |
|  | N | mean | SD | N | mean | SD | N | mean | SD | p-value |
| **Days from biopsy to surgery** |  |  |  |  |  |  |  |  |  |  |
|  | 98664 | 24.17 | 17.56 | 16791 | 37.94 | 24.51 | 595 | 54.84 | 38.48 | 0.00000 |
| **Age at Diagnosis** |  |  |  |  |  |  |  |  |  |  |
|  | 98664 | 75.02 | 6.16 | 16791 | 75.01 | 6.27 | 595 | 74.16 | 6.31 | 0.00370 |
| **% of Zip Code with Less than a High School Education** | | | | | | | | | | |
|  | 98664 | 15.37 | 10.41 | 16791 | 14.17 | 10.21 | 595 | 13.85 | 10.02 | 0.00000 |
| **% of Zip Code Living Below Poverty Line** | | | | | | | | | | |
|  | 98664 | 12.26 | 8.50 | 16791 | 12.38 | 8.55 | 595 | 12.19 | 9.37 | 0.25530 |
| **Charlson Comorbidity Index** | | | | | | | | | | |
|  | 98664 | 0.63 | 1.06 | 16791 | 0.73 | 1.15 | 595 | 0.74 | 1.25 | 0.00000 |
| **Elixhauser Score** | | | | | | | | | | |
|  | 98664 | 1.81 | 5.18 | 16791 | 2.38 | 5.83 | 595 | 3.27 | 7.67 | 0.00000 |
| **Sequence Number of Cancer** | | | | | | | | | | |
|  | 98664 | 1.07 | 0.29 | 16791 | 1.11 | 0.36 | 595 | 1.15 | 0.39 | 0.00000 |
| **Tumor Size (mm)** |  |  |  |  |  |  |  |  |  |  |
|  | 98664 | 18.51 | 18.26 | 16791 | 20.31 | 23.09 | 595 | 23.06 | 19.27 | 0.00000 |
| **Year of Breast Cancer Diagnosis** |  |  |  |  |  |  |  |  |  |  |
|  | 98664 | 2004.79 | 5.53 | 16791 | 2006.48 | 5.17 | 595 | 2005.69 | 5.91 | 0.00000 |
| **Number of Lymph Nodes Positive** |  |  |  |  |  |  |  |  |  |  |
|  | 98664 | 0.84 | 2.65 | 16791 | 1.02 | 2.94 | 595 | 1.47 | 3.67 | 0.00000 |

Supplemental Table 6: Number of unique medical oncologists consulted, categorical demographic and clinical variables

| **Number of unique medical oncologists consulted** | 0 | 0 | 1 | 1 | 2+ | 2+ |  |
| --- | --- | --- | --- | --- | --- | --- | --- |
|  | N | % | N | % | N | % | p-value |
| **Interval (Days)** |  |  |  |  |  |  | 0.00000 |
| 1-30 | 74057 | 75.1 | 7976 | 47.5 | 176 | 29.6 |  |
| 31-60 | 20788 | 21.1 | 6645 | 39.6 | 244 | 41.0 |  |
| 61-90 | 2846 | 2.9 | 1449 | 8.6 | 84 | 14.1 |  |
| 91-120 | 636 | 0.6 | 442 | 2.6 | 38 | 6.4 |  |
| 121-180 | 337 | 0.3 | 279 | 1.7 | 53 | 8.9 |  |
| **Background** |  |  |  |  |  |  | 0.00146 |
| European Ancestry | 87620 | 88.8 | 14709 | 87.6 | 522 | 87.7 |  |
| African Ancestry | 6286 | 6.4 | 1190 | 7.1 | 43 | 7.2 |  |
| Other or Unknown | 1757 | 1.8 | *** | *** | *** | *** |  |
| Asian | 2039 | 2.1 | *** | *** | *** | *** |  |
| Hispanic | 962 | 1.0 | *** | *** | *** | *** |  |
| **Census Region of the United States** |  |  |  |  |  |  | 0.00000 |
| Northeast | 18432 | 18.7 | 2541 | 15.1 | 86 | 14.5 |  |
| Midwest | 15596 | 15.8 | 2909 | 17.3 | 106 | 17.8 |  |
| West | 41599 | 42.2 | 8872 | 52.8 | 327 | 55.0 |  |
| South | 23037 | 23.3 | 2469 | 14.7 | 76 | 12.8 |  |
| **Marital Status** |  |  |  |  |  |  | 0.09523 |
| Not married | 52420 | 53.1 | 9071 | 54.0 | 313 | 52.6 |  |
| Married | 46244 | 46.9 | 7720 | 46.0 | 282 | 47.4 |  |
| **Type of Metropolitan Area** |  |  |  |  |  |  | 0.00000 |
| Big Metro | 53093 | 53.8 | 9762 | 58.1 | 381 | 64.0 |  |
| Metro | 29042 | 29.4 | 4917 | 29.3 | 150 | 25.2 |  |
| Urban | 5830 | 5.9 | 825 | 4.9 | 30 | 5.0 |  |
| Less Urban/Rural | 10699 | 10.8 | 1287 | 7.7 | 34 | 5.7 |  |
| **SEER Defined Sex** |  |  |  |  |  |  | 0.05119 |
| Male | 901 | 0.9 | 157 | 0.9 | 12 | 2.0 |  |
| Female | 97763 | 99.1 | 16634 | 99.1 | 583 | 98.0 |  |
| Histology |  |  |  |  |  |  | 0.00025 |
| Ductal | 85621 | 86.8 | 14363 | 85.5 | 501 | 84.2 |  |
| Lobular | 10852 | 11.0 | 2023 | 12.0 | 78 | 13.1 |  |
| Other or Unknown | 2191 | 2.2 | 405 | 2.4 | 16 | 2.7 |  |
| **Grade** |  |  |  |  |  |  | 0.00000 |
| Well | 23706 | 24.0 | 3784 | 22.5 | 118 | 19.8 |  |
| Moderate | 42619 | 43.2 | 7498 | 44.7 | 267 | 44.9 |  |
| Poor | 23787 | 24.1 | 4397 | 26.2 | 169 | 28.4 |  |
| Undifferentiated | 970 | 1.0 | *** | *** | *** | *** |  |
| Unknown | 7582 | 7.7 | *** | *** | *** | *** |  |
| **ER or PR Positive** |  |  |  |  |  |  | 0.00157 |
| Negative | 20722 | 21.0 | 3327 | 19.8 | 131 | 22.0 |  |
| Positive | 77942 | 79.0 | 13464 | 80.2 | 464 | 78.0 |  |
| **HER2 Positive** |  |  |  |  |  |  | 0.00000 |
| Negative | 96620 | 97.9 | 16210 | 96.5 | 583 | 98.0 |  |
| Positive | 2044 | 2.1 | 581 | 3.5 | 12 | 2.0 |  |
| **AJCC Stage** |  |  |  |  |  |  | 0.00000 |
| Stage 1 | 59008 | 59.8 | 9240 | 55.0 | 280 | 47.1 |  |
| Stage 2 | 33880 | 34.3 | 6126 | 36.5 | 237 | 39.8 |  |
| Stage 3 | 5776 | 5.9 | 1425 | 8.5 | 78 | 13.1 |  |
| **Mastectomy Status** |  |  |  |  |  |  | 0.00000 |
| Lumpectomy Only | 51729 | 52.4 | 8291 | 49.4 | 256 | 43.0 |  |
| Mastectomy | 46935 | 47.6 | 8500 | 50.6 | 339 | 57.0 |  |
| **Were nodes examined** |  |  |  |  |  |  | 0.55799 |
| No | 1724 | 1.7 | 291 | 1.7 | 14 | 2.4 |  |
| Yes | 96940 | 98.3 | 16500 | 98.3 | 581 | 97.6 |  |
| **Reconstructive Surgery** |  |  |  |  |  |  | 0.00000 |
| No | 95798 | 97.1 | 15919 | 94.8 | 550 | 92.4 |  |
| Yes | 2866 | 2.9 | 872 | 5.2 | 45 | 7.6 |  |
| **Adjuvant Chemotherapy** |  |  |  |  |  |  | 0.00000 |
| No Adjuvant | 76099 | 77.1 | 12016 | 71.6 | 391 | 65.7 |  |
| Adjuvant | 22565 | 22.9 | 4775 | 28.4 | 204 | 34.3 |  |
| **Adjuvant Radiotherapy** |  |  |  |  |  |  | 0.16997 |
| No Radiotherapy Adjuvant | 45237 | 45.8 | 7782 | 46.3 | 291 | 48.9 |  |
| Radiotherapy Adjuvant | 53427 | 54.2 | 9009 | 53.7 | 304 | 51.1 |  |

***** used to hide numbers <12 for privacy.**

Supplemental Table 7: Number of unique medical oncologists consulted, continuous demographic and clinical variables after propensity score based weighting

| **Number of unique medical oncologists consulted** | 0 | 0 | 0 | 1 | 1 | 1 | 2+ | 2+ | 2+ |
| --- | --- | --- | --- | --- | --- | --- | --- | --- | --- |
|  | N | mean | SD | N | mean | SD | N | mean | SD |
| **Days from biopsy to surgery (not in model)** | | | | | | | | | |
|  | 98664 | 24.45 | 17.71 | 16791 | 36.36 | 23.87 | 595 | 54.52 | 37.98 |
| **Age at Diagnosis** |  |  |  |  |  |  |  |  |  |
|  | 98664 | 75.02 | 6.18 | 16791 | 75.00 | 6.17 | 595 | 75.32 | 6.30 |
| **% of Zip Code with Less than a High School Education** | | | | | | | | | |
|  | 98664 | 15.19 | 10.40 | 16791 | 15.22 | 10.25 | 595 | 15.58 | 9.89 |
| **% of Zip Code Living Below Poverty Line** | | | | | | | | | |
|  | 98664 | 12.28 | 8.50 | 16791 | 12.26 | 8.43 | 595 | 12.32 | 8.38 |
| **Charlson Comorbidity Index** | | | | | | | | | |
|  | 98664 | 0.65 | 1.08 | 16791 | 0.65 | 1.07 | 595 | 0.63 | 1.05 |
| **Elilxhauser Score** |  |  |  |  |  |  |  |  |  |
|  | 98664 | 1.90 | 5.31 | 16791 | 1.93 | 5.33 | 595 | 2.12 | 5.69 |
| **Sequence Number of Cancer** |  |  |  |  |  |  |  |  |  |
|  | 98664 | 1.08 | 0.30 | 16791 | 1.08 | 0.30 | 595 | 1.07 | 0.29 |
| **Tumor Size** |  |  |  |  |  |  |  |  |  |
|  | 98664 | 18.79 | 18.90 | 16791 | 18.79 | 18.35 | 595 | 19.88 | 16.06 |
| **Year of Breast Cancer Diagnosis** |  |  |  |  |  |  |  |  |  |
|  | 98664 | 2005.04 | 5.51 | 16791 | 2004.95 | 5.48 | 595 | 2004.61 | 5.62 |
| **Number of Lymph Nodes Positive** |  |  |  |  |  |  |  |  |  |
|  | 98664 | 0.87 | 2.71 | 16791 | 0.88 | 2.69 | 595 | 1.06 | 3.04 |

Supplemental Table 8: Number of unique medical oncologists consulted, categorical demographic and clinical variables after propensity score based weighting

| **Number of unique medical oncologists consulted** | 0 | 0 | 1 | 1 | 2+ | 2+ |
| --- | --- | --- | --- | --- | --- | --- |
|  | N | % | N | % | N | % |
| **Interval (Days, not in model)** |  |  |  |  |  |  |
| 1-30 | 74057 | 74.4 | 7976 | 50.9 | 176 | 28.0 |
| 31-60 | 20788 | 21.5 | 6645 | 37.5 | 244 | 42.4 |
| 61-90 | 2846 | 3.0 | 1449 | 7.7 | 84 | 13.9 |
| 91-120 | 636 | 0.7 | 442 | 2.3 | 38 | 7.7 |
| 121-180 | 337 | 0.3 | 279 | 1.5 | 53 | 8.0 |
| **Background** |  |  |  |  |  |  |
| European Ancestry | 87620 | 88.6 | 14709 | 88.5 | 522 | 86.9 |
| African Ancestry | 6286 | 6.5 | 1190 | 6.6 | 43 | 7.5 |
| Other or Unknown | 1757 | 1.8 | *** | *** | *** | *** |
| Asian | 2039 | 2.1 | *** | *** | *** | *** |
| Hispanic | 962 | 1.0 | *** | *** | *** | *** |
| **Census Region of the United States** |  |  |  |  |  |  |
| Northeast | 18432 | 18.1 | 2541 | 18.2 | 86 | 17.8 |
| Midwest | 15596 | 16.0 | 2909 | 15.8 | 106 | 15.7 |
| West | 41599 | 43.8 | 8872 | 43.8 | 327 | 44.8 |
| South | 23037 | 22.0 | 2469 | 22.2 | 76 | 21.6 |
| **Marital Status** |  |  |  |  |  |  |
| Not married | 52420 | 53.3 | 9071 | 53.2 | 313 | 53.1 |
| Married | 46244 | 46.7 | 7720 | 46.8 | 282 | 46.9 |
| **Type of Metropolitan Area** |  |  |  |  |  |  |
| Big Metro | 53093 | 54.5 | 9762 | 53.2 | 381 | 54.0 |
| Metro | 29042 | 29.4 | 4917 | 30.0 | 150 | 29.7 |
| Urban | 5830 | 5.8 | 825 | 5.9 | 30 | 6.8 |
| Less Urban/Rural | 10699 | 10.4 | 1287 | 10.9 | 34 | 9.5 |
| **SEER Defined Sex** |  |  |  |  |  |  |
| Male | 901 | 0.9 | 157 | 1.0 | 12 | 1.0 |
| Female | 97763 | 99.1 | 16634 | 99.0 | 583 | 99.0 |
| **Histology** |  |  |  |  |  |  |
| Ductal | 85621 | 86.6 | 14363 | 86.4 | 501 | 86.3 |
| Lobular | 10852 | 11.2 | 2023 | 11.3 | 78 | 11.1 |
| Other or Unknown | 2191 | 2.3 | 405 | 2.3 | 16 | 2.6 |
| **Grade** |  |  |  |  |  |  |
| Well | 23706 | 23.8 | 3784 | 24.0 | 118 | 20.0 |
| Moderate | 42619 | 43.4 | 7498 | 43.0 | 267 | 44.1 |
| Poor | 23787 | 24.4 | 4397 | 24.5 | 169 | 25.7 |
| Undifferentiated | 970 | 1.0 | *** | *** | *** | *** |
| Unknown | 7582 | 7.4 | *** | *** | *** | *** |
| **ER or PR Positive** |  |  |  |  |  |  |
| Negative | 20722 | 20.8 | 3327 | 21.3 | 131 | 21.7 |
| Positive | 77942 | 79.2 | 13464 | 78.7 | 464 | 78.3 |
| **HER2 Positive** |  |  |  |  |  |  |
| Negative | 96620 | 97.7 | 16210 | 97.8 | 583 | 98.2 |
| Positive | 2044 | 2.3 | 581 | 2.2 | 12 | 1.8 |
| **AJCC Stage** |  |  |  |  |  |  |
| Stage 1 | 59008 | 59.0 | 9240 | 58.8 | 280 | 55.3 |
| Stage 2 | 33880 | 34.7 | 6126 | 34.9 | 237 | 36.8 |
| Stage 3 | 5776 | 6.3 | 1425 | 6.3 | 78 | 7.9 |
| **Mastectomy Status** |  |  |  |  |  |  |
| Lumpectomy Only | 51729 | 51.9 | 8291 | 52.0 | 256 | 53.2 |
| Mastectomy | 46935 | 48.1 | 8500 | 48.0 | 339 | 46.8 |
| **Were nodes examined** |  |  |  |  |  |  |
| No | 1724 | 1.8 | 291 | 1.7 | 14 | 1.9 |
| Yes | 96940 | 98.2 | 16500 | 98.3 | 581 | 98.1 |
| **Reconstructive Surgery** |  |  |  |  |  |  |
| No | 95798 | 96.7 | 15919 | 96.7 | 550 | 96.9 |
| Yes | 2866 | 3.3 | 872 | 3.3 | 45 | 3.1 |
| **Adjuvant Chemotherapy** |  |  |  |  |  |  |
| No Adjuvant | 76099 | 76.3 | 12016 | 76.0 | 391 | 75.8 |
| Adjuvant | 22565 | 23.7 | 4775 | 24.0 | 204 | 24.2 |
| **Adjuvant Radiotherapy** |  |  |  |  |  |  |
| No Radiotherapy Adjuvant | 45237 | 45.9 | 7782 | 45.6 | 291 | 43.8 |
| Radiotherapy Adjuvant | 53427 | 54.1 | 9009 | 54.4 | 304 | 56.2 |

***** used to hide numbers <12 for privacy.**

Supplemental Table 9: Number of unique surgeons consulted, continuous demographic and clinical variables

| **Number of unique surgeons consulted** | 0 | 0 | 0 | 1 | 1 | 1 | 2 | 2 | 2 | 3+ | 3+ | 3+ |  |
| --- | --- | --- | --- | --- | --- | --- | --- | --- | --- | --- | --- | --- | --- |
|  | N | mean | SD | N | mean | SD | N | mean | SD | N | mean | SD | p-value |
| **Days from biopsy to surgery** |  |  |  |  |  |  |  |  |  |  |  |  |  |
|  | 35780 | 19.41 | 15.63 | 73745 | 28.19 | 19.15 | 6016 | 42.08 | 27.14 | 509 | 54.49 | 32.39 | 0.00000 |
| **Age at Diagnosis** |  |  |  |  |  |  |  |  |  |  |  |  |  |
|  | 35780 | 75.20 | 6.21 | 73745 | 75.04 | 6.18 | 6016 | 73.90 | 5.93 | 509 | 72.86 | 5.49 | 0.00000 |
| **% of Zip Code with Less than a High School Education** | | | | | | | | | | | | | |
|  | 35780 | 17.14 | 10.86 | 73745 | 14.36 | 10.04 | 6016 | 13.96 | 10.19 | 509 | 13.04 | 10.46 | 0.00000 |
| **% of Zip Code Living Below Poverty Line** | | | | | | | | | | | | | |
|  | 35780 | 12.05 | 8.66 | 73745 | 12.44 | 8.43 | 6016 | 11.75 | 8.63 | 509 | 11.14 | 8.63 | 0.00000 |
| **Charlson Comorbidity Index** |  |  |  |  |  |  |  |  |  |  |  |  |  |
|  | 35780 | 0.64 | 1.08 | 73745 | 0.66 | 1.07 | 6016 | 0.60 | 1.03 | 509 | 0.60 | 1.07 | 0.00030 |
| **Elixhauser Score** |  |  |  |  |  |  |  |  |  |  |  |  |  |
|  | 35780 | 2.13 | 5.50 | 73745 | 1.81 | 5.21 | 6016 | 1.64 | 5.12 | 509 | 1.51 | 4.97 | 0.00000 |
| **Sequence Number of Cancer** |  |  |  |  |  |  |  |  |  |  |  |  |  |
|  | 35780 | 1.08 | 0.29 | 73745 | 1.08 | 0.31 | 6016 | 1.08 | 0.29 | 509 | 1.05 | 0.24 | 0.00000 |
| **Tumor Size (mm)** |  |  |  |  |  |  |  |  |  |  |  |  |  |
|  | 35780 | 19.64 | 21.49 | 73745 | 18.38 | 17.85 | 6016 | 18.69 | 17.97 | 509 | 19.59 | 15.85 | 0.00000 |
| **Year of Breast Cancer Diagnosis** | |  |  |  |  |  |  |  |  |  |  |  |  |
|  | 35780 | 2002.00 | 5.70 | 73745 | 2006.45 | 4.79 | 6016 | 2005.71 | 5.34 | 509 | 2005.53 | 5.35 | 0.00000 |
| **Number of Lymph Nodes Positive** | | | | | | | | | | | | | |
|  | 35780 | 0.98 | 2.87 | 73745 | 0.81 | 2.58 | 6016 | 0.94 | 3.06 | 509 | 1.05 | 2.80 | 0.00000 |

Supplemental Table 10: Number of unique surgeons consulted, categorical demographic and clinical variables

| **Number of unique surgeons consulted** | 0 | 0 | 1 | 1 | 2 | 2 | 3+ | 3+ |  |
| --- | --- | --- | --- | --- | --- | --- | --- | --- | --- |
|  | N | % | N | % | N | % | N | % | p-value |
| **Interval (Days)** |  |  |  |  |  |  |  |  | 0.00000 |
| 1-30 | 29816 | 83.3 | 49823 | 67.6 | 2456 | 40.8 | 114 | 22.4 |  |
| 31-60 | 5187 | 14.5 | 19720 | 26.7 | 2530 | 42.1 | 240 | 47.2 |  |
| 61-90 | 578 | 1.6 | 3047 | 4.1 | 668 | 11.1 | 86 | 16.9 |  |
| 91-120 | 121 | 0.3 | 750 | 1.0 | 208 | 3.5 | 37 | 7.3 |  |
| 121-180 | 78 | 0.2 | 405 | 0.5 | 154 | 2.6 | 32 | 6.3 |  |
| **Background** |  |  |  |  |  |  |  |  | 0.00000 |
| European Ancestry | 31602 | 88.3 | 65566 | 88.9 | 5239 | 87.1 | 444 | 87.2 |  |
| African Ancestry | 2502 | 7.0 | 4548 | 6.2 | 433 | 7.2 | 36 | 7.1 |  |
| Other or Unknown | 546 | 1.5 | 1401 | 1.9 | *** | *** | *** | *** |  |
| Asian | 739 | 2.1 | 1514 | 2.1 | *** | *** | *** | *** |  |
| Hispanic | 391 | 1.1 | 716 | 1.0 | *** | *** | *** | *** |  |
| **Census Region of the United States** |  |  |  |  |  |  |  |  | 0.00000 |
| Northeast | 6777 | 18.9 | 12763 | 17.3 | 1388 | 23.1 | 131 | 25.7 |  |
| Midwest | 7680 | 21.5 | 10043 | 13.6 | 827 | 13.7 | 61 | 12.0 |  |
| West | 13652 | 38.2 | 34101 | 46.2 | 2782 | 46.2 | 263 | 51.7 |  |
| South | 7671 | 21.4 | 16838 | 22.8 | 1019 | 16.9 | 54 | 10.6 |  |
| **Marital Status** |  |  |  |  |  |  |  |  | 0.00000 |
| Not married | 19584 | 54.7 | 38907 | 52.8 | 3083 | 51.2 | 230 | 45.2 |  |
| Married | 16196 | 45.3 | 34838 | 47.2 | 2933 | 48.8 | 279 | 54.8 |  |
| **Type of Metropolitan Area** |  |  |  |  |  |  |  |  | 0.00000 |
| Big Metro | 19366 | 54.1 | 39662 | 53.8 | 3835 | 63.7 | 373 | 73.3 |  |
| Metro | 9885 | 27.6 | 22692 | 30.8 | 1434 | 23.8 | 98 | 19.3 |  |
| Urban | 2086 | 5.8 | 4295 | 5.8 | *** | *** | *** | *** |  |
| Less Urban/Rural | 4443 | 12.4 | 7096 | 9.6 | *** | *** | *** | *** |  |
| **SEER Defined Sex** |  |  |  |  |  |  |  |  | 0.00000 |
| Male | 429 | 1.2 | 594 | 0.8 | *** | *** | *** | *** |  |
| Female | 35351 | 98.8 | 73151 | 99.2 | *** | *** | *** | *** |  |
| **Histology** |  |  |  |  |  |  |  |  | 0.00000 |
| Ductal | 30965 | 86.5 | 63944 | 86.7 | 5147 | 85.6 | 429 | 84.3 |  |
| Lobular | 3825 | 10.7 | 8301 | 11.3 | 759 | 12.6 | 68 | 13.4 |  |
| Other or Unknown | 990 | 2.8 | 1500 | 2.0 | 110 | 1.8 | 12 | 2.4 |  |
| **Grade** |  |  |  |  |  |  |  |  | 0.00000 |
| Well | 7381 | 20.6 | 18727 | 25.4 | 1393 | 23.2 | 107 | 21.0 |  |
| Moderate | 14638 | 40.9 | 32862 | 44.6 | 2664 | 44.3 | 220 | 43.2 |  |
| Poor | 9216 | 25.8 | 17536 | 23.8 | 1470 | 24.4 | 131 | 25.7 |  |
| Undifferentiated | 463 | 1.3 | 589 | 0.8 | *** | *** | *** | *** |  |
| Unknown | 4082 | 11.4 | 4031 | 5.5 | *** | *** | *** | *** |  |
| **ER or PR Positive** |  |  |  |  |  |  |  |  | 0.00000 |
| Negative | 9033 | 25.2 | 13806 | 18.7 | 1227 | 20.4 | 114 | 22.4 |  |
| Positive | 26747 | 74.8 | 59939 | 81.3 | 4789 | 79.6 | 395 | 77.6 |  |
| **HER2 Positive** |  |  |  |  |  |  |  |  | 0.00000 |
| Negative | 35383 | 98.9 | 71681 | 97.2 | *** | *** | *** | *** |  |
| Positive | 397 | 1.1 | 2064 | 2.8 | *** | *** | *** | *** |  |
| **AJCC Stage** |  |  |  |  |  |  |  |  | 0.00000 |
| Stage 1 | 20351 | 56.9 | 44393 | 60.2 | 3514 | 58.4 | 270 | 53.0 |  |
| Stage 2 | 13203 | 36.9 | 24747 | 33.6 | 2104 | 35.0 | 189 | 37.1 |  |
| Stage 3 | 2226 | 6.2 | 4605 | 6.2 | 398 | 6.6 | 50 | 9.8 |  |
| **Mastectomy Status** |  |  |  |  |  |  |  |  | 0.00000 |
| Lumpectomy Only | 15280 | 42.7 | 41602 | 56.4 | 3145 | 52.3 | 249 | 48.9 |  |
| Mastectomy | 20500 | 57.3 | 32143 | 43.6 | 2871 | 47.7 | 260 | 51.1 |  |
| **Were nodes examined** |  |  |  |  |  |  |  |  | 0.00000 |
| No | 971 | 2.7 | 921 | 1.2 | 124 | 2.1 | 13 | 2.6 |  |
| Yes | 34809 | 97.3 | 72824 | 98.8 | 5892 | 97.9 | 496 | 97.4 |  |
| **Reconstructive Surgery** |  |  |  |  |  |  |  |  | 0.00000 |
| No | 35120 | 98.2 | 71137 | 96.5 | 5579 | 92.7 | 431 | 84.7 |  |
| Yes | 660 | 1.8 | 2608 | 3.5 | 437 | 7.3 | 78 | 15.3 |  |
| **Adjuvant Chemotherapy** |  |  |  |  |  |  |  |  | 0.00000 |
| No Adjuvant | 27912 | 78.0 | 55744 | 75.6 | 4482 | 74.5 | 368 | 72.3 |  |
| Adjuvant | 7868 | 22.0 | 18001 | 24.4 | 1534 | 25.5 | 141 | 27.7 |  |
| **Adjuvant Radiotherapy** |  |  |  |  |  |  |  |  | 0.00000 |
| No Radiotherapy Adjuvant | 19213 | 53.7 | 31079 | 42.1 | 2779 | 46.2 | 239 | 47.0 |  |
| Radiotherapy Adjuvant | 16567 | 46.3 | 42666 | 57.9 | 3237 | 53.8 | 270 | 53.0 |  |

***** used to hide numbers <12 for privacy.**

Supplemental Table 11: Number of unique surgeons consulted, continuous demographic and clinical variables after propensity score based weighting

| **Number of unique surgeons consulted** | 0 | 0 | 0 | 1 | 1 | 1 | 2 | 2 | 2 | 3+ | 3+ | 3+ |
| --- | --- | --- | --- | --- | --- | --- | --- | --- | --- | --- | --- | --- |
|  | N | mean | SD | N | mean | SD | N | mean | SD | N | mean | SD |
| **Days from biopsy to surgery (not in model)** | | |  |  |  |  |  |  |  |  |  |  |
|  | 35780 | 20.76 | 16.33 | 73745 | 27.58 | 19.14 | 6016 | 40.64 | 26.83 | 509 | 55.79 | 33.40 |
| **Age at Diagnosis** | | | |  |  |  |  |  |  |  |  |  |
|  | 35780 | 75.03 | 6.19 | 73745 | 75.01 | 6.19 | 6016 | 75.08 | 6.18 | 509 | 74.95 | 6.02 |
| **% of Zip Code with Less than a High School Education** | | | | |  |  |  |  |  |  |  |  |
|  | 35780 | 15.22 | 10.35 | 73745 | 15.24 | 10.45 | 6016 | 15.40 | 10.49 | 509 | 15.87 | 10.15 |
| **% of Zip Code Living Below Poverty Line** | | | | | |  |  |  |  |  |  |  |
|  | 35780 | 12.31 | 8.41 | 73745 | 12.28 | 8.55 | 6016 | 12.34 | 8.57 | 509 | 12.67 | 8.90 |
| **Charlson Comorbidity Index** |  |  |  |  |  |  |  |  |  |  |  |  |
|  | 35780 | 0.64 | 1.07 | 73745 | 0.65 | 1.07 | 6016 | 0.67 | 1.09 | 509 | 0.74 | 1.13 |
| **Elixhauser Score** |  |  |  |  |  |  |  |  |  |  |  |  |
|  | 35780 | 1.87 | 5.27 | 73745 | 1.89 | 5.28 | 6016 | 1.98 | 5.39 | 509 | 2.27 | 5.50 |
| **Sequence Number of Cancer** |  |  |  |  |  |  |  |  |  |  |  |  |
|  | 35780 | 1.08 | 0.30 | 73745 | 1.08 | 0.30 | 6016 | 1.08 | 0.30 | 509 | 1.07 | 0.28 |
| **Tumor Size** |  |  |  |  |  |  |  |  |  |  |  |  |
|  | 35780 | 18.97 | 19.44 | 73745 | 18.93 | 19.96 | 6016 | 18.80 | 17.86 | 509 | 18.66 | 14.85 |
| **Year of Breast Cancer Diagnosis** | |  |  |  |  |  |  |  |  |  |  |  |
|  | 35780 | 2005.02 | 5.52 | 73745 | 2004.98 | 5.57 | 6016 | 2004.96 | 5.53 | 509 | 2004.86 | 5.37 |
| **Number of Lymph Nodes Positive** | | | | |  |  |  |  |  |  |  |  |
|  | 35780 | 0.87 | 2.70 | 73745 | 0.87 | 2.71 | 6016 | 0.85 | 2.63 | 509 | 0.76 | 2.43 |

Supplemental Table 12: Number of unique surgeons consulted, categorical demographic and clinical variables after propensity score based weighting

| **Number of unique surgeons consulted** | 0 | 0 | 1 | 1 | 2 | 2 | 3+ | 3+ |
| --- | --- | --- | --- | --- | --- | --- | --- | --- |
|  | N | % | N | % | N | % | N | % |
| **Interval (Days, not in model)** |  |  |  |  |  |  |  |  |
| 1-30 | 29816 | 80.5 | 49823 | 69.0 | 2456 | 43.8 | 114 | 21.6 |
| 31-60 | 5187 | 16.9 | 19720 | 25.5 | 2530 | 40.2 | 240 | 47.6 |
| 61-90 | 578 | 2.0 | 3047 | 3.9 | 668 | 10.4 | 86 | 16.1 |
| 91-120 | 121 | 0.4 | 750 | 1.0 | 208 | 3.1 | 37 | 7.8 |
| 121-180 | 78 | 0.3 | 405 | 0.6 | 154 | 2.4 | 32 | 6.9 |
| **Background** |  |  |  |  |  |  |  |  |
| European Ancestry | 31602 | 88.8 | 65566 | 88.7 | 5239 | 88.4 | 444 | 86.7 |
| African Ancestry | 2502 | 6.4 | 4548 | 6.5 | 433 | 6.7 | 36 | 8.5 |
| Other or Unknown | 546 | 1.8 | 1401 | 1.8 | *** | *** | *** | *** |
| Asian | 739 | 2.1 | 1514 | 2.1 | *** | *** | *** | *** |
| Hispanic | 391 | 1.0 | 716 | 1.0 | *** | *** | *** | *** |
| **Census Region of the United States** |  |  |  |  |  |  |  |  |
| Northeast | 6777 | 18.2 | 12763 | 18.3 | 1388 | 18.6 | 131 | 19.8 |
| Midwest | 7680 | 15.9 | 10043 | 16.1 | 827 | 15.6 | 61 | 16.2 |
| West | 13652 | 43.5 | 34101 | 43.5 | 2782 | 43.6 | 263 | 42.1 |
| South | 7671 | 22.4 | 16838 | 22.1 | 1019 | 22.2 | 54 | 21.8 |
| **Marital Status** |  |  |  |  |  |  |  |  |
| Not married | 19584 | 53.1 | 38907 | 53.3 | 3083 | 53.5 | 230 | 56.1 |
| Married | 16196 | 46.9 | 34838 | 46.7 | 2933 | 46.5 | 279 | 43.9 |
| **Type of Metropolitan Area** |  |  |  |  |  |  |  |  |
| Big Metro | 19366 | 53.3 | 39662 | 54.3 | 3835 | 54.0 | 373 | 54.6 |
| Metro | 9885 | 29.7 | 22692 | 29.3 | 1434 | 29.5 | 98 | 27.2 |
| Urban | 2086 | 6.1 | 4295 | 5.8 | *** | *** | *** | *** |
| Less Urban/Rural | 4443 | 10.9 | 7096 | 10.6 | *** | *** | *** | *** |
| **SEER Defined Sex** |  |  |  |  |  |  |  |  |
| Male | 429 | 1.0 | 594 | 0.9 | *** | *** | *** | *** |
| Female | 35351 | 99.0 | 73151 | 99.1 | *** | *** | *** | *** |
| **Histology** |  |  |  |  |  |  |  |  |
| Ductal | 30965 | 86.6 | 63944 | 86.5 | 5147 | 86.7 | 429 | 87.0 |
| Lobular | 3825 | 11.1 | 8301 | 11.2 | 759 | 11.0 | 68 | 9.9 |
| Other or Unknown | 990 | 2.3 | 1500 | 2.3 | 110 | 2.2 | 12 | 3.1 |
| **Grade** |  |  |  |  |  |  |  |  |
| Well | 7381 | 23.6 | 18727 | 23.7 | 1393 | 23.6 | 107 | 25.7 |
| Moderate | 14638 | 43.2 | 32862 | 43.2 | 2664 | 43.4 | 220 | 39.9 |
| Poor | 9216 | 24.7 | 17536 | 24.5 | 1470 | 24.6 | 131 | 26.0 |
| Undifferentiated | 463 | 1.0 | 589 | 1.0 | *** | *** | *** | *** |
| Unknown | 4082 | 7.5 | 4031 | 7.7 | *** | *** | *** | *** |
| **ER or PR Positive** |  |  |  |  |  |  |  |  |
| Negative | 9033 | 20.9 | 13806 | 20.9 | 1227 | 21.0 | 114 | 22.6 |
| Positive | 26747 | 79.1 | 59939 | 79.1 | 4789 | 79.0 | 395 | 77.4 |
| **HER2 Positive** |  |  |  |  |  |  |  |  |
| Negative | 35383 | 97.8 | 71681 | 97.7 | *** | *** | *** | *** |
| Positive | 397 | 2.2 | 2064 | 2.3 | *** | *** | *** | *** |
| **AJCC Stage** |  |  |  |  |  |  |  |  |
| Stage 1 | 20351 | 58.7 | 44393 | 58.9 | 3514 | 59.2 | 270 | 59.8 |
| Stage 2 | 13203 | 34.9 | 24747 | 34.8 | 2104 | 34.5 | 189 | 33.6 |
| Stage 3 | 2226 | 6.4 | 4605 | 6.3 | 398 | 6.3 | 50 | 6.6 |
| **Mastectomy Status** |  |  |  |  |  |  |  |  |
| Lumpectomy Only | 15280 | 52.0 | 41602 | 51.7 | 3145 | 51.9 | 249 | 51.3 |
| Mastectomy | 20500 | 48.0 | 32143 | 48.3 | 2871 | 48.1 | 260 | 48.7 |
| **Were nodes examined** |  |  |  |  |  |  |  |  |
| No | 971 | 1.8 | 921 | 1.9 | 124 | 1.7 | 13 | 2.0 |
| Yes | 34809 | 98.2 | 72824 | 98.1 | 5892 | 98.3 | 496 | 98.0 |
| **Reconstructive Surgery** |  |  |  |  |  |  |  |  |
| No | 35120 | 96.8 | 71137 | 96.7 | 5579 | 96.6 | 431 | 96.9 |
| Yes | 660 | 3.2 | 2608 | 3.3 | 437 | 3.4 | 78 | 3.1 |
| **Adjuvant Chemotherapy** |  |  |  |  |  |  |  |  |
| No Adjuvant | 27912 | 76.1 | 55744 | 76.3 | 4482 | 76.4 | 368 | 77.3 |
| Adjuvant | 7868 | 23.9 | 18001 | 23.7 | 1534 | 23.6 | 141 | 22.7 |
| **Adjuvant Radiotherapy** |  |  |  |  |  |  |  |  |
| No Radiotherapy Adjuvant | 19213 | 45.7 | 31079 | 46.2 | 2779 | 46.1 | 239 | 42.4 |
| Radiotherapy Adjuvant | 16567 | 54.3 | 42666 | 53.8 | 3237 | 53.9 | 270 | 57.6 |

***** used to hide numbers <12 for privacy.**

Supplemental Figure 1: New Patient Encounters. A. Breast Cancer Specific Mortality Estimates Using Competing Risk Regression Results. B. Subdistribution Hazard Ratios from Breast Cancer Specific Competing Risk Regressions. Extreme delay interval values beyond which there are fewer than 12 cases within each group are marked by parentheses. The median within the group is marked by a vertical bar. C. Boxplot Showing Distribution of Delay Interval in Days by New Patient Encounters. Small numbers within whiskers are hidden by a box to protect privacy. D. Overall Survival Estimates Using Cox Regression Results E. Overall Survival Hazard Ratios from Cox Regressions.


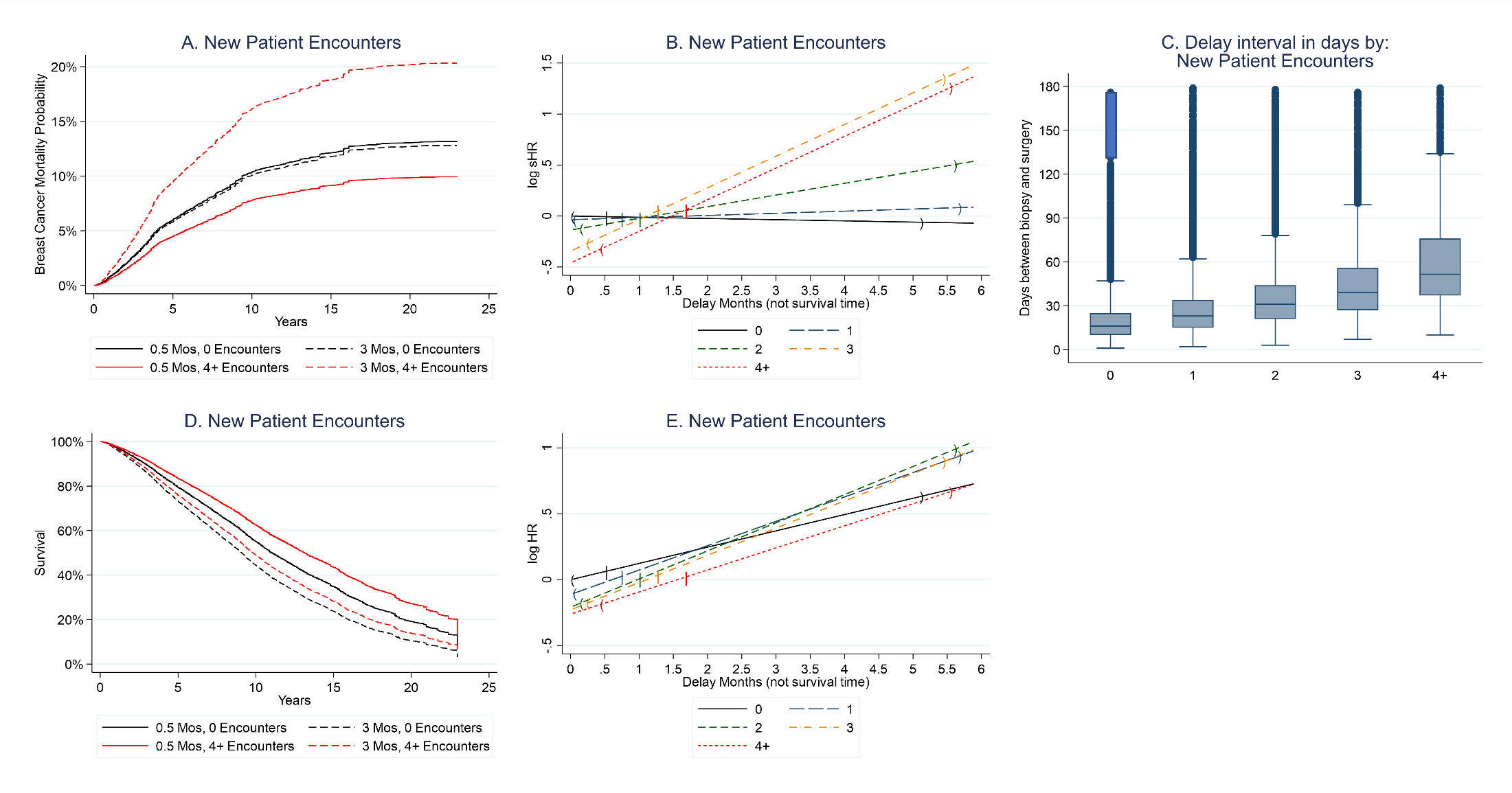


Supplemental Table 13. Estimates akin to those presented in Table 3 for: New Patient Encounters.

| **Delay Effects per Month for New Patient Encounters.** | | | | | | | | | |
| --- | --- | --- | --- | --- | --- | --- | --- | --- | --- |
|  | Breast Cancer Mortality | | | | | Overall Survival | | | |
|  | N | HR or Day | 95% CI | 95% CI | P-value | HR or Day | 95% CI | 95% CI | P-value |
| HR by Month for New Patient Encounters=0 | 49213 | 0.9883 | 0.9186 | 1.0632 | 0.7516 | 1.1315 | 1.0942 | 1.1702 | 0.0000 |
| HR by Month for New Patient Encounters=1 | 43138 | 1.0210 | 0.9558 | 1.0908 | 0.5366 | 1.2032 | 1.1710 | 1.2362 | 0.0000 |
| HR by Month for New Patient Encounters=2 | 17114 | 1.1214 | 1.0278 | 1.2235 | 0.0100 | 1.2380 | 1.1912 | 1.2866 | 0.0000 |
| HR by Month for New Patient Encounters=3 | 4947 | 1.3640 | 1.2005 | 1.5498 | 0.0000 | 1.2294 | 1.1257 | 1.3427 | 0.0000 |
| HR by Month for New Patient Encounters=4+ | 1638 | 1.3639 | 1.1326 | 1.6425 | 0.0011 | 1.1822 | 1.0522 | 1.3283 | 0.0049 |
| Interaction HR for New Patient Encounters=0 vs 1 |  |  |  |  | 0.5136 |  |  |  | 0.0039 |
| Interaction HR for New Patient Encounters=0 vs 2 |  |  |  |  | 0.0291 |  |  |  | 0.0004 |
| Interaction HR for New Patient Encounters=0 vs 3 |  |  |  |  | 0.0000 |  |  |  | 0.0809 |
| Interaction HR for New Patient Encounters=0 vs 4+ |  |  |  |  | 0.0015 |  |  |  | 0.4719 |
| HR at baseline delay for New Patient Encounters=0 vs 1 |  | 0.9653 | 0.8857 | 1.0520 | 0.4207 | 0.8942 | 0.8619 | 0.9277 | 0.0000 |
| HR at baseline delay for New Patient Encounters=0 vs 2 |  | 0.8716 | 0.7607 | 0.9987 | 0.0479 | 0.8121 | 0.7655 | 0.8614 | 0.0000 |
| HR at baseline delay for New Patient Encounters=0 vs 3 |  | 0.7090 | 0.5462 | 0.9203 | 0.0098 | 0.7959 | 0.6922 | 0.9150 | 0.0013 |
| HR at baseline delay for New Patient Encounters=0 vs 4+ |  | 0.6311 | 0.3738 | 1.0653 | 0.0849 | 0.7705 | 0.5773 | 1.0283 | 0.0766 |
| Day log HR=0 for New Patient Encounters=1 |  | 32.9524 | -23.7816 | 89.6864 | 0.2550 | 55.4505 | 29.7128 | 81.1882 | 0.0000 |
| Day log HR=0 for New Patient Encounters=2 |  | 33.0984 | 13.5256 | 52.6713 | 0.0009 | 70.4881 | 43.8318 | 97.1444 | 0.0000 |
| Day log HR=0 for New Patient Encounters=3 |  | 32.4872 | 16.5218 | 48.4525 | 0.0001 | 83.7519 | 26.6133 | 140.8905 | 0.0041 |
| Day log HR=0 for New Patient Encounters=4+ |  | 43.4891 | 12.3190 | 74.6592 | 0.0062 | 181.1931 | -154.8087 | 517.1950 | 0.2905 |

Supplemental Figure 2: Number of unique medical oncologists consulted. A. Breast Cancer Specific Mortality Estimates Using Competing Risk Regression Results. B. Subdistribution Hazard Ratios from Breast Cancer Specific Competing Risk Regressions. Extreme delay interval values beyond which there are fewer than 12 cases within each group are marked by parentheses. The median within the group is marked by a vertical bar. C. Boxplot Showing Distribution of Delay Interval in Days by New Patient Encounters. Small numbers within whiskers are hidden by a box to protect privacy. D. Overall Survival Estimates Using Cox Regression Results E. Overall Survival Hazard Ratios from Cox Regressions.

Supplemental Table 14. Estimates akin to those presented in Table 3 for number of unique medical oncologists consulted.

| **Delay Effects per Month for Number of Unique Medical Oncologists Consulted.** | | | | | | | | | |
| --- | --- | --- | --- | --- | --- | --- | --- | --- | --- |
|  | Breast Cancer Mortality | | | | | Overall Survival | | | |
|  | N | HR or Day | 95% CI | 95% CI | P-value | HR or Day | 95% CI | 95% CI | P-value |
| HR by month, number unique medical oncologists  consulted=0 | 98664 | 0.9265 | 0.8852 | 0.9698 | 0.0010 | 1.0606 | 1.0372 | 1.0846 | 0.0000 |
| HR by month for number of unique medical oncologists  consulted=1 | 16791 | 1.1576 | 1.0762 | 1.2451 | 0.0001 | 1.1956 | 1.1560 | 1.2365 | 0.0000 |
| HR by month for number of unique medical oncologists  consulted=2+ | 595 | 1.3522 | 1.1332 | 1.6134 | 0.0008 | 1.1819 | 1.0299 | 1.3564 | 0.0173 |
| Interaction HR for number of unique medical oncologists  consulted=0 vs 1 |  |  |  |  | 0.0000 |  |  |  | 0.0000 |
| Interaction HR for number of unique medical oncologists  consulted=0 vs 2+ |  |  |  |  | 0.0001 |  |  |  | 0.1197 |
| HR at baseline delay for number of unique medical  oncologists consulted=0 vs 1 |  | 0.8069 | 0.7170 | 0.9080 | 0.0004 | 0.8508 | 0.8081 | 0.8958 | 0.0000 |
| HR at baseline delay for number of unique medical  oncologists consulted=0 vs 2+ |  | 0.6229 | 0.3868 | 1.0029 | 0.0514 | 0.7885 | 0.6036 | 1.0300 | 0.0813 |
| Day log HR=0 for number of unique medical  oncologists consulted=1 |  | 29.3329 | 20.0348 | 38.6311 | 0.0000 | 41.0607 | 32.8661 | 49.2553 | 0.0000 |
| Day log HR=0 for number of unique medical  oncologists consulted=2+ |  | 38.1155 | 11.5949 | 64.6361 | 0.0048 | 66.7782 | 15.5053 | 118.0511 | 0.0107 |

Supplemental Figure 3: Number of surgeons consulted. A. Breast Cancer Specific Mortality Estimates Using Competing Risk Regression Results. B. Subdistribution Hazard Ratios from Breast Cancer Specific Competing Risk Regressions. Extreme delay interval values beyond which there are fewer than 12 cases within each group are marked by parentheses. The median within the group is marked by a vertical bar. C. Boxplot Showing Distribution of Delay Interval in Days by New Patient Encounters. Small numbers within whiskers are hidden by a box to protect privacy. D. Overall Survival Estimates Using Cox Regression Results E. Overall Survival Hazard Ratios from Cox Regressions.

Supplemental Table 15. Estimates akin to those presented in Table 3 for number of unique surgeons consulted.

| Delay Effects per Month for Number of Unique Surgeons Consulted. | | | | | | | | | |
| --- | --- | --- | --- | --- | --- | --- | --- | --- | --- |
|  | Breast Cancer Mortality | | | | | Overall Survival | | | |
|  | N | HR or Day | 95% CI | 95% CI | P-value | HR or Day | 95% CI | 95% CI | P-value |
| HR by month for Number of unique surgeons consulted=0 | 35780 | 0.9577 | 0.8875 | 1.0334 | 0.2658 | 1.0872 | 1.0507 | 1.1249 | 0.0000 |
| HR by month for Number of unique surgeons consulted=1 | 73745 | 1.0965 | 1.0411 | 1.1548 | 0.0005 | 1.1954 | 1.1685 | 1.2230 | 0.0000 |
| HR by month for Number of unique surgeons consulted=2 | 6016 | 1.1290 | 1.0011 | 1.2734 | 0.0480 | 1.1994 | 1.1399 | 1.2620 | 0.0000 |
| HR by month for Number of unique surgeons consulted=3+ | 509 | 1.0001 | 0.7644 | 1.3085 | 0.9992 | 1.4451 | 1.2472 | 1.6744 | 0.0000 |
| Interaction HR for Number of unique surgeons consulted=0 vs 1 |  |  |  |  | 0.0037 |  |  |  | 0.0000 |
| Interaction HR for Number of unique surgeons consulted=0 vs 2 |  |  |  |  | 0.0233 |  |  |  | 0.0013 |
| Interaction HR for Number of unique surgeons consulted=0 vs 3+ |  |  |  |  | 0.7609 |  |  |  | 0.0002 |
| HR at baseline delay for Number of unique surgeons consulted=0 vs 1 |  | 0.8780 | 0.8097 | 0.9522 | 0.0017 | 0.8798 | 0.8496 | 0.9111 | 0.0000 |
| HR at baseline delay for Number of unique surgeons consulted=0 vs 2 |  | 0.8360 | 0.6827 | 1.0238 | 0.0832 | 0.7833 | 0.7196 | 0.8527 | 0.0000 |
| HR at baseline delay for Number of unique surgeons consulted=0 vs 3+ |  | 0.7436 | 0.3928 | 1.4078 | 0.3630 | 0.4749 | 0.3308 | 0.6817 | 0.0001 |
| Day log HR=0 for Number of unique surgeons consulted=1 |  | 29.2594 | 17.3358 | 41.1830 | 0.0000 | 41.0516 | 30.2954 | 51.8078 | 0.0000 |
| Day log HR=0 for Number of unique surgeons consulted=2 |  | 33.1222 | 11.5532 | 54.6912 | 0.0026 | 75.6285 | 45.3372 | 105.9199 | 0.0000 |
| Day log HR=0 for Number of unique surgeons consulted=3+ |  | 207.9926 | -834.04 | 1250.0218 | 0.6956 | 79.6377 | 56.7733 | 102.5020 | 0.0000 |

Supplemental Figure 4: Number of unique radiation oncologists consulted. A. Breast Cancer Specific Mortality Estimates Using Competing Risk Regression Results. B. Subdistribution Hazard Ratios from Breast Cancer Specific Competing Risk Regressions. Extreme delay interval values beyond which there are fewer than 12 cases within each group are marked by parentheses. The median within the group is marked by a vertical bar. C. Boxplot Showing Distribution of Delay Interval in Days by New Patient Encounters. Small numbers within whiskers are hidden by a box to protect privacy. D. Overall Survival Estimates Using Cox Regression Results E. Overall Survival Hazard Ratios from Cox Regressions.

Supplemental Table 16. Estimates akin to those presented in Table 3 for number of unique radiation oncologists consulted.

| Delay Effects per Month for Number of Unique Radiation Oncologists Consulted. | | | | | | | | | |
| --- | --- | --- | --- | --- | --- | --- | --- | --- | --- |
|  | Breast Cancer Mortality | | | | | Overall Survival | | | |
|  | N | HR or Day | 95% CI | 95% CI | P-value | HR or Day | 95% CI | 95% CI | P-value |
| HR by month for Number of unique radiation oncologists consulted=0 | 103888 | 1.0462 | 0.9997 | 1.0948 | 0.0514 | 1.1509 | 1.1182 | 1.1845 | 0.0000 |
| HR by month for Number of unique radiation oncologists consulted=1 | 11948 | 1.1416 | 0.9920 | 1.3137 | 0.0646 | 1.2142 | 1.1483 | 1.2838 | 0.0000 |
| HR by month for Number of unique radiation oncologists consulted=2+ | 214 | 0.9949 | 0.5060 | 1.9560 | 0.9881 | 1.2352 | 0.9756 | 1.5640 | 0.0793 |
| Interaction HR for Number of unique radiation oncologists consulted=0 vs 1 |  |  |  |  | 0.2395 |  |  |  | 0.0644 |
| Interaction HR for Number of unique radiation oncologists consulted=0 vs 2+ |  |  |  |  | 0.8819 |  |  |  | 0.5491 |
| HR at baseline delay for Number of unique radiation oncologists consulted=0 vs 1 |  | 0.8688 | 0.7164 | 1.0537 | 0.1532 | 0.8412 | 0.7810 | 0.9061 | 0.0000 |
| HR at baseline delay for Number of unique radiation oncologists consulted=0 vs 2+ |  | 1.1097 | 0.2383 | 5.1669 | 0.8945 | 0.5772 | 0.3420 | 0.9742 | 0.0396 |
| Day log HR=0 for Number of unique radiation oncologists consulted=1 |  | 49.0516 | 8.3931 | 89.7101 | 0.0181 | 98.2775 | 24.3541 | 172.2009 | 0.0092 |
| Day log HR=0 for Number of unique radiation oncologists consulted=2+ |  | 62.9689 | -492.3 | 618.2386 | 0.8241 | 236.4122 | -392.10 | 864.9249 | 0.4610 |

Supplemental Figure 5: Established physician encounters. A. Breast Cancer Specific Mortality Estimates Using Competing Risk Regression Results. B. Subdistribution Hazard Ratios from Breast Cancer Specific Competing Risk Regressions. Extreme delay interval values beyond which there are fewer than 12 cases within each group are marked by parentheses. The median within the group is marked by a vertical bar. C. Boxplot Showing Distribution of Delay Interval in Days by New Patient Encounters. Small numbers within whiskers are hidden by boxes to protect privacy. D. Overall Survival Estimates Using Cox Regression Results E. Overall Survival Hazard Ratios from Cox Regressions.


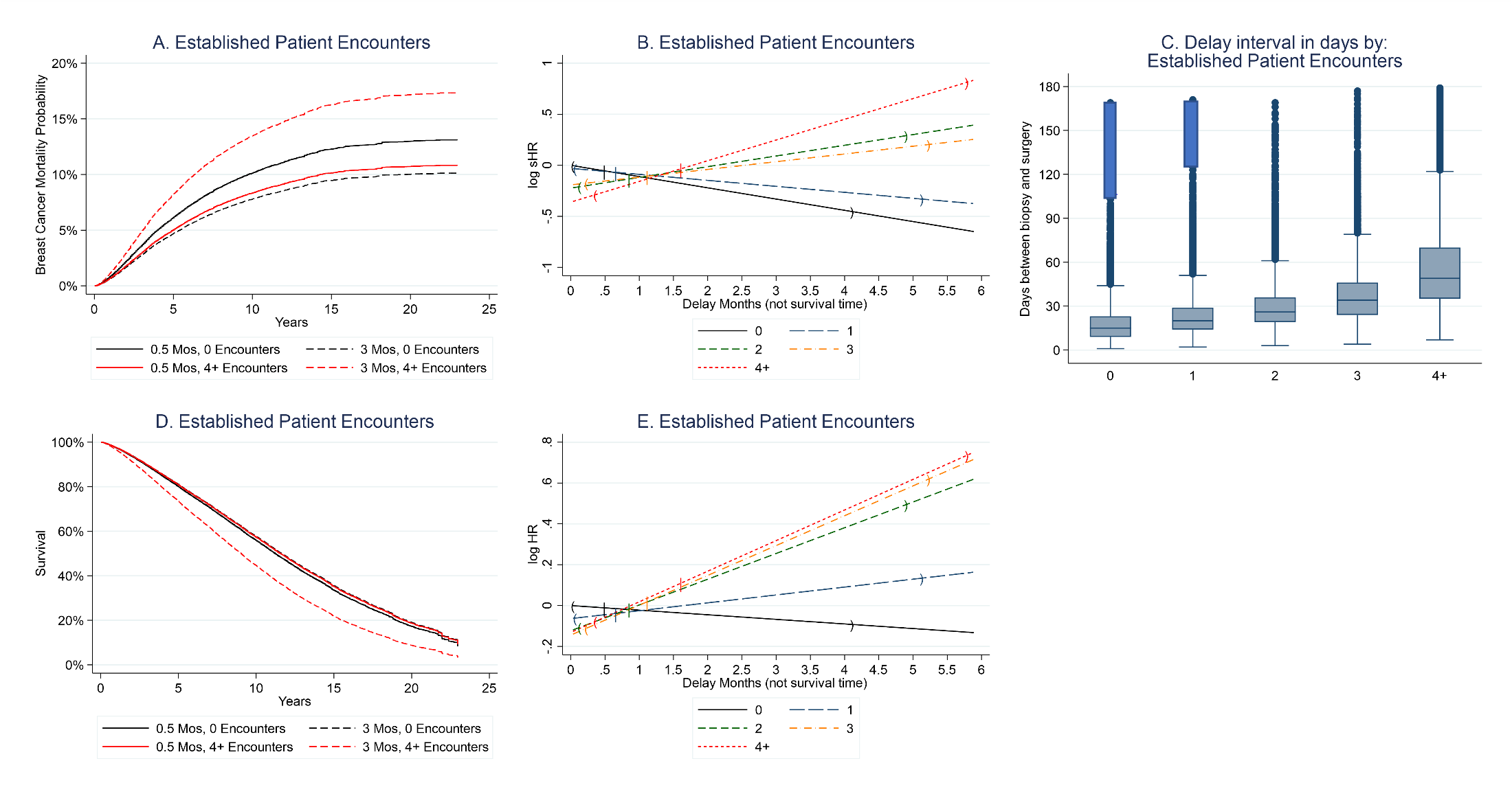


Supplemental Table 17. Estimates akin to those presented in Table 3 for established patient encounters.

| Delay Effects per Month for Number of Established Patient Encounters. | | | | | | | | | |
| --- | --- | --- | --- | --- | --- | --- | --- | --- | --- |
|  | Breast Cancer Mortality | | | | | Overall Survival | | | |
|  | N | HR or Day | 95% CI | 95% CI | P-value | HR or Day | 95% CI | 95% CI | P-value |
| HR by month for Established Patient Encounters=0 | 36969 | 0.8957 | 0.8048 | 0.9968 | 0.0434 | 0.9778 | 0.9343 | 1.0233 | 0.3326 |
| HR by month for Established Patient Encounters=1 | 38779 | 0.9430 | 0.8659 | 1.0270 | 0.1777 | 1.0394 | 1.0022 | 1.0779 | 0.0378 |
| HR by month for Established Patient Encounters=2 | 21555 | 1.1105 | 1.0050 | 1.2271 | 0.0396 | 1.1344 | 1.0872 | 1.1836 | 0.0000 |
| HR by month for Established Patient Encounters=3 | 9861 | 1.0786 | 0.9534 | 1.2203 | 0.2293 | 1.1574 | 1.0912 | 1.2276 | 0.0000 |
| HR by month for Established Patient Encounters=4+ | 8886 | 1.2248 | 1.1160 | 1.3442 | 0.0000 | 1.1617 | 1.1054 | 1.2209 | 0.0000 |
| Interaction HR for Established Patient Encounters=0 vs 1 |  |  |  |  | 0.4598 |  |  |  | 0.0391 |
| Interaction HR for Established Patient Encounters=0 vs 2 |  |  |  |  | 0.0040 |  |  |  | 0.0000 |
| Interaction HR for Established Patient Encounters=0 vs 3 |  |  |  |  | 0.0257 |  |  |  | 0.0000 |
| Interaction HR for Established Patient Encounters=0 vs 4+ |  |  |  |  | 0.0000 |  |  |  | 0.0000 |
| HR at baseline delay, Established Patient Encounters=0 vs 1 |  | 0.9704 | 0.8795 | 1.0707 | 0.5493 | 0.9382 | 0.8993 | 0.9787 | 0.0031 |
| HR at baseline delay, Established Patient Encounters=0 vs 2 |  | 0.8000 | 0.7021 | 0.9116 | 0.0008 | 0.8842 | 0.8363 | 0.9348 | 0.0000 |
| HR at baseline delay, Established Patient Encounters=0 vs 3 |  | 0.8255 | 0.6803 | 1.0017 | 0.0520 | 0.8654 | 0.7901 | 0.9478 | 0.0019 |
| HR at baseline delay, Established Patient Encounters=0 vs 4+ |  | 0.6972 | 0.5438 | 0.8940 | 0.0045 | 0.8768 | 0.7796 | 0.9861 | 0.0282 |
| Day log HR=0 for Established Patient Encounters=1 |  | 17.7420 | -12.6334 | 48.1173 | 0.2523 | 31.7977 | 14.6917 | 48.9038 | 0.0003 |
| Day log HR=0 for Established Patient Encounters=2 |  | 31.5861 | 19.8464 | 43.3258 | 0.0000 | 25.2180 | 18.6553 | 31.7808 | 0.0000 |
| Day log HR=0 for Established Patient Encounters=3 |  | 31.4017 | 13.0988 | 49.7045 | 0.0008 | 26.0962 | 16.6053 | 35.5871 | 0.0000 |
| Day log HR=0 for Established Patient Encounters=4+ |  | 35.0661 | 18.6286 | 51.5037 | 0.0000 | 23.2212 | 8.2900 | 38.1525 | 0.0023 |

Supplemental Figure 6: Number of encounters. A. Breast Cancer Specific Mortality Estimates Using Competing Risk Regression Results. B. Subdistribution Hazard Ratios from Breast Cancer Specific Competing Risk Regressions. Extreme delay interval values beyond which there are fewer than 12 cases within each group are marked by parentheses. The median within the group is marked by a vertical bar. C. Boxplot Showing Distribution of Delay Interval in Days by New Patient Encounters. Small numbers within whiskers are hidden by boxes to protect privacy. D. Overall Survival Estimates Using Cox Regression Results E. Overall Survival Hazard Ratios from Cox Regressions.
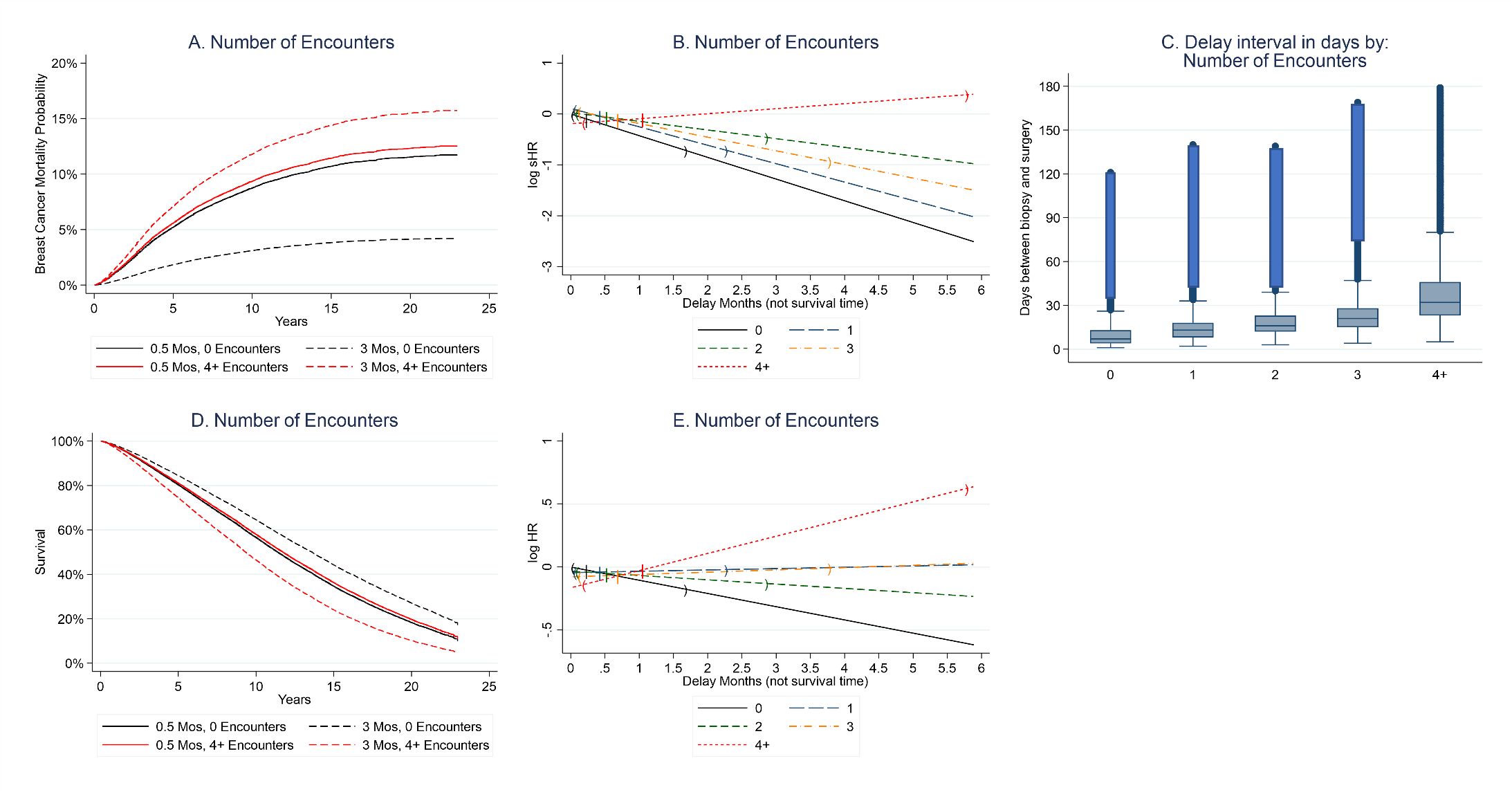


Supplemental Table 18. Estimates akin to those presented in Table 3 for number of encounters any type.

| Delay Effects per Month for Number of Encounters of Any Type. | | | | | | | | | |
| --- | --- | --- | --- | --- | --- | --- | --- | --- | --- |
|  | Breast Cancer Mortality | | | | | Overall Survival | | | |
|  | N | HR or Day | 95% CI | 95% CI | P-value | HR or Day | 95% CI | 95% CI | P-value |
| HR by month for Number of Encounters=0 | 7356 | 0.6531 | 0.3844 | 1.1095 | 0.1151 | 0.9003 | 0.7315 | 1.1081 | 0.3215 |
| HR by month for Number of Encounters=1 | 17047 | 0.6963 | 0.5600 | 0.8658 | 0.0011 | 1.0107 | 0.9272 | 1.1017 | 0.8091 |
| HR by month for Number of Encounters=2 | 21794 | 0.8432 | 0.7062 | 1.0067 | 0.0592 | 0.9664 | 0.8993 | 1.0386 | 0.3526 |
| HR by month for Number of Encounters=3 | 19443 | 0.7650 | 0.6515 | 0.8984 | 0.0011 | 1.0184 | 0.9563 | 1.0845 | 0.5699 |
| HR by month for Number of Encounters=4+ | 50410 | 1.1034 | 1.0522 | 1.1572 | 0.0001 | 1.1462 | 1.1211 | 1.1718 | 0.0000 |
| Interaction HR for Number of Encounters=0 vs 1 |  |  |  |  | 0.8263 |  |  |  | 0.3134 |
| Interaction HR for Number of Encounters=0 vs 2 |  |  |  |  | 0.3701 |  |  |  | 0.5272 |
| Interaction HR for Number of Encounters=0 vs 3 |  |  |  |  | 0.5754 |  |  |  | 0.2655 |
| Interaction HR for Number of Encounters=0 vs 4+ |  |  |  |  | 0.0534 |  |  |  | 0.0234 |
| HR at baseline delay for Number of Encounters=0 vs 1 |  | 1.1162 | 0.8883 | 1.4025 | 0.3455 | 0.9565 | 0.8780 | 1.0421 | 0.3090 |
| HR at baseline delay for Number of Encounters=0 vs 2 |  | 1.0274 | 0.8165 | 1.2928 | 0.8177 | 0.9675 | 0.8880 | 1.0542 | 0.4509 |
| HR at baseline delay for Number of Encounters=0 vs 3 |  | 1.0834 | 0.8566 | 1.3702 | 0.5040 | 0.9253 | 0.8473 | 1.0104 | 0.0837 |
| HR at baseline delay for Number of Encounters=0 vs 4+ |  | 0.8256 | 0.6679 | 1.0204 | 0.0762 | 0.8480 | 0.7839 | 0.9173 | 0.0000 |
| Day log HR=0 for Number of Encounters=1 |  | -52.1571 | -612.4193 | 508.1051 | 0.8552 | 11.7029 | -2.3423 | 25.7482 | 0.1024 |
| Day log HR=0 for Number of Encounters=2 |  | -3.2197 | -36.7180 | 30.2787 | 0.8506 | 14.1691 | -12.0349 | 40.3732 | 0.2892 |
| Day log HR=0 for Number of Encounters=3 |  | -15.4018 | -110.1383 | 79.3348 | 0.7500 | 19.1805 | -2.2386 | 40.5997 | 0.0792 |
| Day log HR=0 for Number of Encounters=4+ |  | 11.1233 | 3.9984 | 18.2483 | 0.0022 | 20.7836 | 8.7671 | 32.8000 | 0.0007 |

Supplemental Figure 7: Number of biopsies. A. Breast Cancer Specific Mortality Estimates Using Competing Risk Regression Results. B. Subdistribution Hazard Ratios from Breast Cancer Specific Competing Risk Regressions. Extreme delay interval values beyond which there are fewer than 12 cases within each group are marked by parentheses. The median within the group is marked by a vertical bar. C. Boxplot Showing Distribution of Delay Interval in Days by New Patient Encounters. Small numbers within whiskers are hidden by a box to protect privacy. D. Overall Survival Estimates Using Cox Regression Results E. Overall Survival Hazard Ratios from Cox Regressions.


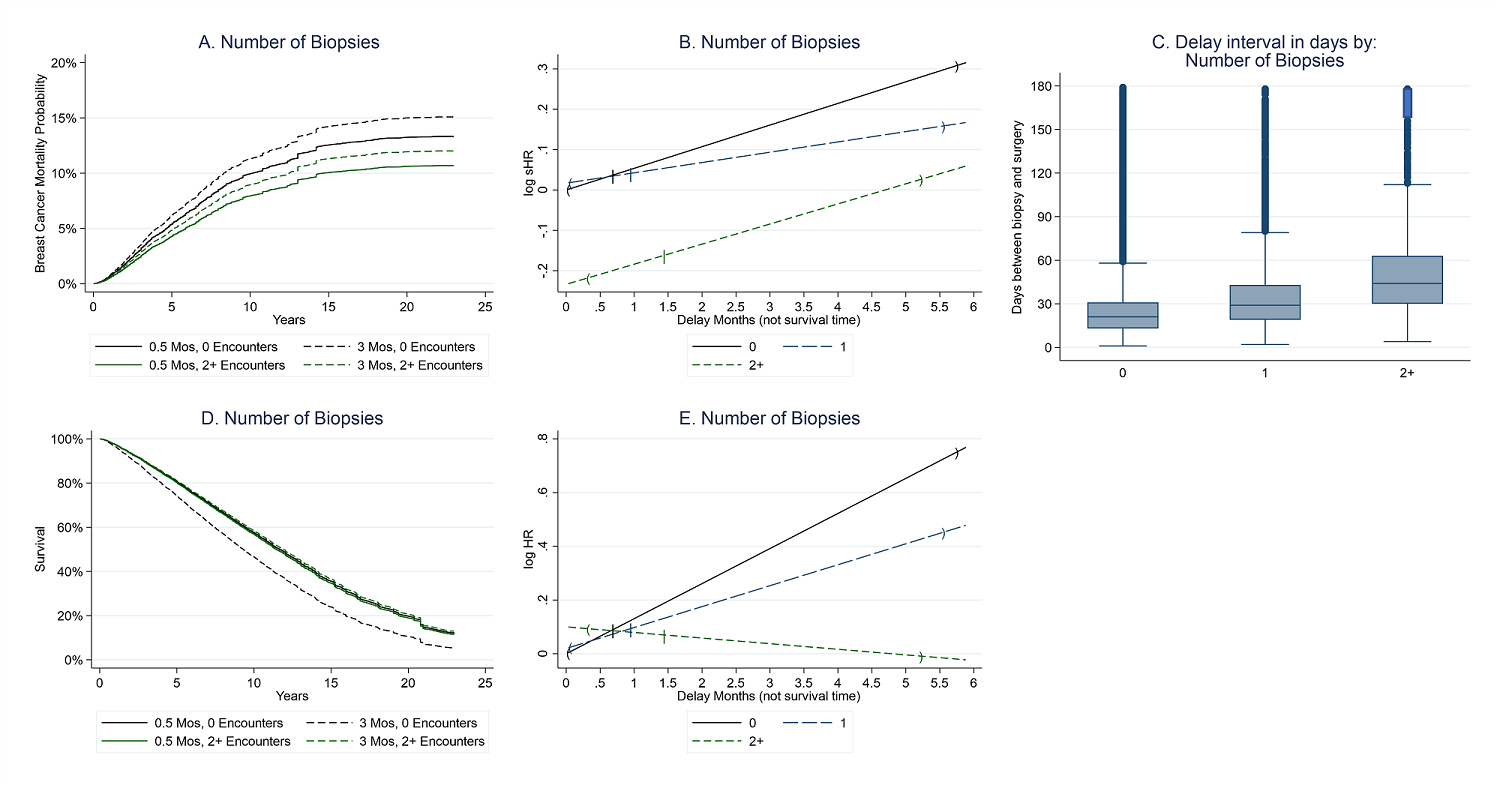


Supplemental Table 19. Estimates akin to those presented in Table 3 for number of biopsies.

| Delay Effects per Month for Number of Biopsies. | | | | | | | | | |
| --- | --- | --- | --- | --- | --- | --- | --- | --- | --- |
|  | Breast Cancer Mortality | | | | | Overall Survival | | | |
|  | N | HR or Day | 95% CI | 95% CI | P-value | HR or Day | 95% CI | 95% CI | P-value |
| HR by month for Number of Biopsies=0 | 98924 | 1.0551 | 1.0125 | 1.0994 | 0.0107 | 1.1395 | 1.1176 | 1.1619 | 0.0000 |
| HR by month for Number of Biopsies=1 | 15362 | 1.0259 | 0.9389 | 1.1208 | 0.5720 | 1.0811 | 1.0415 | 1.1221 | 0.0000 |
| HR by month for Number of Biopsies=2+ | 1764 | 1.0511 | 0.8566 | 1.2897 | 0.6332 | 0.9794 | 0.8794 | 1.0907 | 0.7044 |
| Interaction HR for Number of Biopsies=0 vs 1 |  |  |  |  | 0.5696 |  |  |  | 0.0103 |
| Interaction HR for Number of Biopsies=0 vs 2+ |  |  |  |  | 0.9716 |  |  |  | 0.0065 |
| HR at baseline delay for Number of Biopsies=0 vs 1 |  | 1.0172 | 0.9059 | 1.1420 | 0.7734 | 1.0199 | 0.9711 | 1.0712 | 0.4302 |
| HR at baseline delay for Number of Biopsies=0 vs 2+ |  | 0.7915 | 0.5259 | 1.1912 | 0.2622 | 1.1054 | 0.9183 | 1.3307 | 0.2895 |
| Day log HR=0 for Number of Biopsies=1 |  | 18.4226 | -61.1486 | 97.9938 | 0.6500 | 11.4107 | -10.4156 | 33.2370 | 0.3055 |
| Day log HR=0 for Number of Biopsies=2+ |  | -1.87e+03 | -1.08e+05 | 1.04e+05 | 0.9724 | 20.1451 | -6.2769 | 46.5672 | 0.1351 |

Supplemental Figure 8: Number of imaging studies. A. Breast Cancer Specific Mortality Estimates Using Competing Risk Regression Results. B. Subdistribution Hazard Ratios from Breast Cancer Specific Competing Risk Regressions. Extreme delay interval values beyond which there are fewer than 12 cases within each group are marked by parentheses. The median within the group is marked by a vertical bar. C. Boxplot Showing Distribution of Delay Interval in Days by New Patient Encounters. D. Overall Survival Estimates Using Cox Regression Results E. Overall Survival Hazard Ratios from Cox Regressions.

Supplemental Table 20. Estimates akin to those presented in Table 3 for number of imaging studies.

| Delay Effects per Month for Number of Imaging Studies | | | | | | | | | |
| --- | --- | --- | --- | --- | --- | --- | --- | --- | --- |
|  | Breast Cancer Mortality | | | | | Overall Survival | | | |
|  | N | HR or Day | 95% CI | 95% CI | P-value | HR or Day | 95% CI | 95% CI | P-value |
| HR by month for Number of Images=0 | 77804 | 0.9053 | 0.8546 | 0.9589 | 0.0007 | 1.1231 | 1.0964 | 1.1504 | 0.0000 |
| HR by month for Number of Images=1 | 25684 | 1.0353 | 0.9612 | 1.1151 | 0.3601 | 1.1492 | 1.1114 | 1.1883 | 0.0000 |
| HR by month for Number of Images=2 | 8791 | 1.0456 | 0.9413 | 1.1614 | 0.4055 | 1.1524 | 1.0924 | 1.2157 | 0.0000 |
| HR by month for Number of Images=3+ | 3771 | 1.1172 | 0.9262 | 1.3475 | 0.2465 | 1.1754 | 1.0339 | 1.3363 | 0.0135 |
| Interaction HR for Number of Images=0 vs 1 |  |  |  |  | 0.0049 |  |  |  | 0.2507 |
| Interaction HR for Number of Images=0 vs 2 |  |  |  |  | 0.0180 |  |  |  | 0.3738 |
| Interaction HR for Number of Images=0 vs 3+ |  |  |  |  | 0.0384 |  |  |  | 0.4999 |
| HR at baseline delay for Number of Images=0 vs 1 |  | 0.9466 | 0.8590 | 1.0431 | 0.2676 | 0.9824 | 0.9414 | 1.0253 | 0.4161 |
| HR at baseline delay for Number of Images=0 vs 2 |  | 1.0312 | 0.8691 | 1.2237 | 0.7246 | 0.9567 | 0.8786 | 1.0418 | 0.3087 |
| HR at baseline delay for Number of Images=0 vs 3+ |  | 1.0397 | 0.6988 | 1.5469 | 0.8476 | 0.9177 | 0.7141 | 1.1795 | 0.5026 |
| Day log HR=0 for Number of Images=1 |  | 12.4573 | -2.9994 | 27.9140 | 0.1142 | 23.4581 | -9.5071 | 56.4233 | 0.1631 |
| Day log HR=0 for Number of Images=2 |  | -6.4967 | -47.1966 | 34.2032 | 0.7544 | 52.2286 | -14.9541 | 119.4112 | 0.1276 |
| Day log HR=0 for Number of Images=3+ |  | -5.6383 | -67.5684 | 56.2918 | 0.8584 | 57.3745 | -32.5117 | 147.2606 | 0.2109 |

Supplemental Figure 9: Number of unique physicians consulted as new patient visits (medical oncologists, surgeons, or radiation oncologists only). A. Breast Cancer Specific Mortality Estimates Using Competing Risk Regression Results. B. Subdistribution Hazard Ratios from Breast Cancer Specific Competing Risk Regressions. Extreme delay interval values beyond which there are fewer than 12 cases within each group are marked by parentheses. The median within the group is marked by a vertical bar. C. Boxplot Showing Distribution of Delay Interval in Days by New Patient Encounters. Small numbers within whiskers are hidden by a box to protect privacy. D. Overall Survival Estimates Using Cox Regression Results E. Overall Survival Hazard Ratios from Cox Regressions.

Supplemental Table 21. Estimates akin to those presented in Table 3 for unique physicians consulted as new patient visits (medical oncologists, surgeons, or radiation oncologists only).

| Delay Effects per Month for Unique Physicians Consulted as New Patient Visits | | | | | | | | | |
| --- | --- | --- | --- | --- | --- | --- | --- | --- | --- |
|  | Breast Cancer Mortality | | | | | Overall Survival | | | |
|  | N | HR or Day | 95% CI | 95% CI | P-value | HR or Day | 95% CI | 95% CI | P-value |
| HR by month for Unique Physicians as New Patient visits=0 | 60933 | 1.0219 | 0.9648 | 1.0823 | 0.4606 | 1.1485 | 1.1171 | 1.1808 | 0.0000 |
| HR by month for Unique Physicians as New Patient Visits=1 | 39887 | 1.0869 | 1.0175 | 1.1610 | 0.0133 | 1.2074 | 1.1717 | 1.2441 | 0.0000 |
| HR by month for Unique Physicians Consulted as New Patient Visits=2 | 11649 | 1.2401 | 1.1167 | 1.3771 | 0.0001 | 1.3010 | 1.2432 | 1.3614 | 0.0000 |
| HR by month for Unique Physicians as New Patient Visits=3 | 3145 | 1.3821 | 1.1069 | 1.7256 | 0.0043 | 1.2945 | 1.1489 | 1.4584 | 0.0000 |
| HR by month for Unique Physicians as New Patient Visits=4+ | 436 | 1.5407 | 1.0414 | 2.2793 | 0.0305 | 1.5354 | 1.2730 | 1.8519 | 0.0000 |
| Interaction HR for Unique Physicians as New Patient Visits=0 vs 1 |  |  |  |  | 0.1582 |  |  |  | 0.0093 |
| Interaction HR for Unique Physicians as New Patient Visits=0 vs 2 |  |  |  |  | 0.0013 |  |  |  | 0.0000 |
| Interaction HR for Unique Physicians as New Patient Visits=0 vs 3 |  |  |  |  | 0.0101 |  |  |  | 0.0549 |
| Interaction HR for Unique Physicians as New Patient Visits=0 vs 4+ |  |  |  |  | 0.0402 |  |  |  | 0.0027 |
| HR at baseline delay for Unique Physicians as New Patient Visits=0 vs 1 |  | 0.9264 | 0.8486 | 1.0112 | 0.0872 | 0.8682 | 0.8357 | 0.9021 | 0.0000 |
| HR at baseline delay for Unique Physicians as New Patient Visits=0 vs 2 |  | 0.7582 | 0.6394 | 0.8990 | 0.0014 | 0.7464 | 0.6932 | 0.8037 | 0.0000 |
| HR at baseline delay for Unique Physicians as New Patient Visits=0 vs 3 |  | 0.8420 | 0.5475 | 1.2947 | 0.4333 | 0.7009 | 0.5592 | 0.8785 | 0.0020 |
| HR at baseline delay for Unique Physicians as New Patient Visits=0 vs 4+ |  | 0.3558 | 0.1188 | 1.0653 | 0.0648 | 0.2940 | 0.1700 | 0.5084 | 0.0000 |
| Day log HR=0 for Unique Physicians as New Patient Visits=1 |  | 37.7284 | 7.6956 | 67.7612 | 0.0138 | 86.1064 | 37.5849 | 134.6278 | 0.0005 |
| Day log HR=0 for Unique Physicians as New Patient Visits=2 |  | 43.5303 | 27.6418 | 59.4189 | 0.0000 | 71.4284 | 53.2751 | 89.5818 | 0.0000 |
| Day log HR=0 for Unique Physicians as New Patient Visits=3 |  | 17.3387 | -16.8593 | 51.5367 | 0.3204 | 90.4550 | 32.2436 | 148.6664 | 0.0023 |
| Day log HR=0 for Unique Physicians as New Patient Visits=4+ |  | 76.6020 | 32.0792 | 121.1248 | 0.0007 | 128.3497 | 83.1511 | 173.5483 | 0.0000 |

**Appendix**. SAS formats used to identify treatments and physician encounters.

PROC FORMAT ;

VALUE $breastcpt /*This includes HCPCS codes */

"96400", "96401", "96402", "96405", "96406", "96408", "96409", "96410", "96411", "96412", "96413", "96414", "96415", "96416", "96417", "96420", "96422", "96423", "96425", "96440", "96445", "96450", "96520", "96521", "96522", "96523", "96530", "96542", "96545", "96549", "99601", "99602", "Q0083", "Q0084", "Q0085", "G0355", "G0356", "G0357", "G0358", "G0359", "G0360", "G0361", "G0362", "G0363", "G9021", "G9022", "G9023", "G9024", "G9025", "G9026", "G9027", "G9028", "G9029", "G9030", "G9031", "G9032", "J0897", "J2430", "J3487", "J3488", "J8510", "J8520", "J8521", "J8530", "J8560", "J8561", "J8562", "J8610", "J8999", "J9000", "J9001", "J9035", "J9045", "J9060", "J9062", "J9070", "J9080", "J9090", "J9091", "J9092", "J9093", "J9094", "J9095", "J9096", "J9097", "J9150", "J9151", "J9165", "J9170", "J9171", "J9178", "J9179", "J9181", "J9182", "J9190", "J9201", "J9202", "J9206", "J9207", "J9211", "J9217", "J9218", "J9219", "J9250", "J9260", "J9264", "J9265", "J9280", "J9290", "J9291", "J9293", "J9328", "J9340", "J9355", "J9390", "J9395", "J9999"="chemocpt" /*Chemotherapy*/

"77371"-"77373","77401", "77402", "77403", "77404", "77406", "77407", "77408", "77409", "77411", "77412", "77413", "77414", "77416", "77417", "77418", "77427", "77431", "77470", "77499", "77520", "77522", "77523", "77525", "77750", "77761", "77762", "77763", "77776", "77777", "77778", "77781", "77782", "77783", "77784", "77789", "77790", "77799", "C9726" = "radiotherapycpt" /*Radiotherapy HCPCS codes */

"19240", "19307", "19220", "19306", "19200", "19305" = "mastwnodescpt" /*Mastectomy HCPCS codes with node examination*/

"19162", "19302" = "lumpwnodescpt" /* Lumpectomy HCPSC codes with node examination*/

"19180", "19303", "19182", "19304" = "mastectomycpt" /* Mastectomy HCPCS codes */

"19160", "19301", "19125", "19126" = "lumpectomycpt" /* Lumpectomy HCPCS codes */

"19101", "19110", "19290", "19291", "76096", "76097", "G8876", "G8877" = "excisbiopcpt" /* Excisional biopsy HCPCS codes. Used with lymph node evaluation to determine if this code represents definitive treatment in addition to a biopsy */

"19120" = "Y:biop19120cpt" /* Biopsy HCPCS codes */

"38500", "38525", "38530" = "lnexcisioncpt" /*Lymph node excision codes used to identify definitive surgical therapy */

"38740", "38745" = "axillarycpt" /* Axillary node dissection used to identify definitive surgical therapy */

"38792", "38900", "G8878" = "sentnodedyecpt" /* Sentinel node HCPCS codes used to identify definitive surgical therapy */

"38790", "78195", "A9520", "A9512", "A9541" = "sentnoderadiocpt" /* Additional codes used to identify lymph node dissection related to definitive surgical therapy */

"10021", "10022" = "fineneedaspcpt" /* Fine needle aspiration (biopsy) */

"19100", "19102", "19103" = "coreneedlecpt" /* Core needle biopsy */

"76095", "76942", "76943", "76355", "77011", "G8946" = "needbiopunspeccpt" /* Biopsy */

"77031", "77032", "77021", "76360", "77012", "G8875" = "biopunspeccpt" /* Biopsy codes */

"99211"-"99215" = "estpatenccpt" /*Established patient visit codes */

"99201"-"99205", "99241"-"99245" = "newpatenccpt" /* New patient visit codes */

"76086", "76087", "76088", "76089", "77053", "77054", "19030" = "ductogramcpt" /* Imaging study codes */

"78811", "78812", "78813", "78814", "78815", "78816", "78890", "78891", "78999", "G0253", "G0254", "G0235", "PI", "PS","G0252" = "petscancpt" /* Imaging study code */

"76092", "77057", "G0202", "G0203", "76083", "77052", "76091", "77056", "G0204", "G0205", "76090", "77055", "G0206", "G0207", "76082", "77051", "76085", "G0236" = "premammcpt" /* Imaging study codes */

"76645", "76880", "76881", "76682" = "ultrasoundcpt" /* Imaging study codes */

"76904", "76903", "77059", "77058", "76376", "76377", "C8903", "C8904","C8905","C8906","C8907","C8908" = "breastmricpt" /* Imaging study codes */

"70450", "70460", "70470", "70480", "70481", "70482", "70486", "70487", "70488", "70490", "70491", "70492", "71250", "71260", "71270", "72125", "72126", "72127", "72128", "72129", "72130", "72131", "72132", "72133", "72192", "72193", "72194", "73200", "73201", "73202", "73700", "73701", "73702", "74150", "74160", "74170", "74176", "74177", "74178", "76497" = "comptomcpt" /* Imaging study codes */

"78300", "78305", "78306", "78315", "78399" = "bonescancpt" /* Imaging study codes */

"70551", "70552", "70553" = "brainmricpt" /* Imaging study codes */

"93762", "93740" = "otherimagecpt" /* Imaging study codes */ ;

VALUE $breasticdproc

"9925" = "chemoicd" /* Chemotherapy ICD 9 code */

"9221","9222","9227","9228","9229", "924","9241","922", "923", "9230", "9321", "9233", "9239" = "radiotherapyicd" /* Radiotherapy ICD-9 code */

"8545", "8546", "8547", "8548", "8543", "8544" = "mastwnodesicd" /*Mastectomy with node*/

"8540", "8541", "8542", "8534", "8536" = "mastectomyicd" /*Mastectomy ICD-9 codes */

"8523", "842" = "lumpectomyicd" /* Lumpectomy ICD-9 codes */

"850","8512" = "excisbiopicd" /*Excisional biopsy iCD-9 codes */

"852", "8520","8521","8522", "8524", "8525" = "Y:lumpORexcis" /* Lumpectomy or excisional biospy codes */

"4023", "4022", "403", "4011", "4029" = "lnexcisionicd" /* Lymph node excision codes */

"4050", "405", "4051" = "axillaryicd" /*Axillary dissection ICD-9 codes */

"8511" = "needbiopunspecicd" /* Biopsy ICD-9 codes */

"4019" = "sentnodedyeicd" /* Sentinel node dye ICD-9 codes */

"8519" = "biopdyecode8519" /* Biopsy ICD-9 codes */

"9216" = "sentnoderadioicd" /* Sentinel node examination code */

"9211", "9212", "9218", "9219" = "petscanicd" /*Imaging ICD-9 codes */

"8591" = "fineneedaspicd" /* Biopsy code */

"8737", "8736" = "premammicd" /* Imaging study code */

"8873" = "ultrasoundicd" /* Imaging study code */

"8897" = "breastmriicd" /* Imaging study code */

"8703", "8741", "8801", "8771", "8838" = "comptomicd" /* Imaging study code */

"9214" = "bonescanicd" /* Imaging study code */

"8891" = "brainmriicd" /* Imaging study code */

"8885" = "otherimageicd" /* Imaging study code */;

VALUE $breasticddiag

"V5811", "V672" "V662", "V581" = "chemodiag" /* Chemotherapy ICD-9 diagnosis codes */

"V580", "V661", "V671" = "radiotherapydiag" /* Radiotherapy ICD-9 diagnosis codes */;

VALUE $revenue

"0331", "0332", "0335", "331", "332", "335" = "chemorev" /* Chemotherapy revenue center*/

"0330", "0333", "330", "333" = "radiotherapyrev" /* Radiotherapy revenue center */;

**HCFA Specialty codes used to identify specialties**

Surgeon HCFA codes: hcfaspec="91" or hcfaspec="02" or hcfaspec="49"

Medical oncology HCFA codes: hcfaspec="82" or hcfaspec="83" or hcfaspec="90"

Radiation Oncology HCFA codes: hcfaspec="92" or there is a relevant radiation therapy visit code on the claim along with the following HCFA codes: hcfaspec="94" or hcfaspec="31" or hcfaspec="30" or hcfaspec="32" or hcfaspec="36" or hcfaspec="70" or hcfaspec=="74"
